# Targeting CXCL1 chemokine signaling for treating cisplatin ototoxicity

**DOI:** 10.3389/fimmu.2023.1125948

**Published:** 2023-03-31

**Authors:** Raheem F. H. Al Aameri, Entkhab M. A. Alanisi, Adu Oluwatosin, Dheyaa Al Sallami, Sandeep Sheth, Ian Alberts, Shree Patel, Leonard P. Rybak, Vickram Ramkumar

**Affiliations:** ^1^ Department of Pharmacology, Southern Illinois University School of Medicine, Springfield, IL, United States; ^2^ Department of Biology, Mustansiriyah University College of Science, Baghdad, Iraq; ^3^ Department of Pharmaceutical Sciences, Larkin University College of Pharmacy, Miami, FL, United States; ^4^ Medical Microbiology, Immunology and Cell Biology (MMICB), Southern Illinois University School of Medicine, Springfield, IL, United States; ^5^ Department of Surgery, Southern Illinois University School of Medicine, Springfield, IL, United States

**Keywords:** Cisplatin, CXCL1, CXCR2, SB225002, Hair cells

## Abstract

Cisplatin is chemotherapy used for solid tumor treatment like lung, bladder, head and neck, ovarian and testicular cancers. However, cisplatin-induced ototoxicity limits the utility of this agent in cancer patients, especially when dose escalations are needed. Ototoxicity is associated with cochlear cell death through DNA damage, the generation of reactive oxygen species (ROS) and the consequent activation of caspase, glutamate excitotoxicity, inflammation, apoptosis and/or necrosis. Previous studies have demonstrated a role of CXC chemokines in cisplatin ototoxicity. In this study, we investigated the role of CXCL1, a cytokine which increased in the serum and cochlea by 24 h following cisplatin administration. Adult male Wistar rats treated with cisplatin demonstrated significant hearing loss, assessed by auditory brainstem responses (ABRs), hair cell loss and loss of ribbon synapse. Immunohistochemical studies evaluated the levels of CXCL1 along with increased presence of CD68 and CD45-positive immune cells in cochlea. Increases in CXCL1 was time-dependent in the spiral ganglion neurons and organ of Corti and was associated with progressive increases in CD45, CD68 and IBA1-positive immune cells. Trans-tympanic administration of SB225002, a chemical inhibitor of CXCR2 (receptor target for CXCL1) reduced immune cell migration, protected against cisplatin-induced hearing loss and preserved hair cell integrity. We show that SB225002 reduced the expression of *CXCL1*, *NOX3*, *iNOS*, *TNF-α*, *IL-6* and *COX-2*. Similarly, knockdown of *CXCR2* by trans-tympanic administration of *CXCR2* siRNA protected against hearing loss and loss of outer hair cells and reduced ribbon synapses. In addition, SB225002 reduced the expression of inflammatory mediators induced by cisplatin. These results implicate the CXCL1 chemokine as an early player in cisplatin ototoxicity, possibly by initiating the immune cascade, and indicate that CXCR2 is a relevant target for treating cisplatin ototoxicity.

## Introduction

Cisplatin (cis-diamminedichloroplatinum II) is a major chemotherapeutic agent used in the treatment of a different solid tumors such as head, neck and ovarian cancers (1). However, cisplatin chemotherapy results in side effects including nephrotoxicity (2) and ototoxicity (3). Studies have reported the cytotoxic mechanisms of cisplatin including DNA damage (4, 5), production of reactive oxygen species (ROS) (6), cytoplasmic caspase activation (7) and mitochondrial dysfunction (8). Cisplatin ototoxicity involves damage or loss of the cells in the organ of Corti, stria vascularis and spiral ganglion neurons, mediated in part by ROS generation (9). Oxidative stress can produce lipid peroxidation, inflammation, DNA damage, and apoptosis or necrosis of cells in the cochlea (10).

Inflammation is being studied as an important component of hearing loss (11). Pro-inflammatory cytokines can stimulate spiral ligament fibrocytes and increase production or activation of inflammatory response mediators such as interleukin 6 (IL-6), tumor necrosis factor-α (TNF- α), chemokines such as CXCL1 and macrophage inflammatory peptide 2 (MIP-2), soluble intercellular adhesion molecule-1 (sICAM-1) and vascular endothelial growth factor (VEGF) (12, 13). These inflammatory mediators activate neutrophil migration to the sites of damage within the cochlea and initiate the inflammatory response, leading to either immune resolution or cell apoptosis and cochlear dysfunction (14). Cytokines are major mediators of cisplatin-induced ototoxicity (15).

Macrophages play an important role in the initiation, maintenance and resolution of the immune responses in the cochlea (16). Activation of cochlear CX3CR1 and CD45-positive macrophages has been demonstrated in the spiral ligament following noise exposure (17). Resident macrophages and supporting cells of the inner ear are implicated in this early immune response activation (16, 18). In addition, a recent study indicated the expression of immune cell markers on resident cells of the organ of Corti, which include supporting cells, and to a lesser extent, sensory cells (19). The close proximity of sensory and supporting cells suggest that stress signals from the former could be readily communicated to the latter to induce inflammatory mediators (20).

Chemokines have been shown to participate actively in the immune response in the cochlea which contributes to hearing loss (21). These include CCL2, CCL4 and CXCL12, which are induced within 6 to 24 h after damage to the cochlea (22, 23) and presumably derive from activation of resident macrophages in the cochlea. Subsequent migration of macrophages and monocytes into the cochlea occurs within 3-4 days which are CX3CR1 and CD45 positive (10, 24). The role of the chemokines, such as CXCL1 and CXCL2, in the recruitment of neutrophils to the site of inflammation or tissue damage has been well studied (25, 26). These chemokines are released by resident immune cells in response to “danger” molecules and serve as homing signals for neutrophil migration to the site of injury. In fact, these chemokines serve as early participants in the inflammatory process (26).

A preliminary cytokine array of the cochlea following cisplatin administration indicate that CXCL1 is increased by 24 h. In this study, we show that cisplatin produces rapid induction of *CXCL1* gene by resident cells in the cochlea. We further examined the role of these chemokines and chemokines receptors as mediators of cochlea inflammation and as potential therapeutic targets for treating cisplatin-induced hearing loss.

## Materials and methods

### Drug and reagents

SB225002 was purchased from TOCRIS bioscience (#2725/10). Antibodies used in this study were purchased from different companies: anti-CtBP2 mouse IgG1 was purchased from BD Biosciences (#612044), Rabbit polyclonal Myosin-VIIa was obtained from Proteus Biosciences (#25-6790), Rabbit polyclonal IgG CXCL1 (ab269939), Rabbit monoclonal IBA1 (ab178846) and Mouse monoclonal IgG1 CD68 (ab31630) were purchased from Abcam, Mouse monoclonal IgG2a CD45 (05-1410) and anti-glutamate receptor 2 (GluR2) IgG2a were purchased from Millipore (#MAB397). Rhodamine (TRITC) AffiniPure Donkey Anti-Rabbit IgG secondary antibody was purchased from Jackson ImmunoResearch Laboratories (711-025-152). Secondary antibodies used were as follows: Alexa FluorTM 568 goat anti-mouse IgG1 (A-21124), Alexa FluorTM 488 goat anti-mouse IgG2a (A-21131) and Alexa FluorTM 647 donkey anti-goat IgG (A-21447) were purchased from Life Technologies. All primers were purchase from integrated DNA technologies (IDT DNA) (Iowa. USA). Normal Goat serum was purchased from SouthernBiotech (0060-01) (Alabama. USA).

### Animal procedure

Male Wistar rats from Envigo (Indianapolis, IN, USA) weighing 200-250 gm were housed in the Division of Laboratory Animal Medicine (DLAM) facility of SIU School of Medicine. Rats were given free access to food and water and were housed in temperature-controlled room with a 12 h light/dark cycle. The total number of animals used in this project was 108 rats. Each group consist from 4-6 animals. All animal experiments were approved and monitored by Southern Illinois University School of Medicine, Laboratory Animal Care and Use Committee (LACUC). Animals were anesthetized with mixture of ketamine (90 g/kg) and xylazine (17mg/kg), administered intraperitoneally and the depth of anesthesia was determined by the absence of reflex to toe pinch within the duration of the experiment. If the depth of anesthesia was insufficient, rats were injected an additional dose of the mixture. Auditory brainstem responses (ABRs) were performed while animal was anesthetized in sound proof chamber. SB225002, siCXR2, scramble or vehicle were delivered *via* trans-tympanic injection into middle ear while cisplatin (11 mg/kg) or equivalent volumes of vehicle were administrated intraperitoneally and post ABRs were recorded 72 h later. Rats were then anesthetized using the same protocol mentioned previously prior to euthanization. One cochlea from each animal was fixed by perfusion of 4% paraformaldehyde for immunohistochemistry studies while the other cochlea was perfused with RNA Later (Thermo Fisher Scientific, Carlsbad CA).

### Auditory brainstem responses

Auditory evoked potentials were recorded as described previously (27). Briefly, rats were anesthetized and placed in double-wall sound proof chamber and stainless steel electrodes were positioned as follow, subdermal electrodes were inserted in the hind flank muscles regions (ground), the positive electrode was inserted between two ears at vertex in the skull and negative electrodes were inserted under the pinna of each ear. Sound stimuli were provided by earphones placed into the ear and acoustic stimuli were generated and applied as tone bursts at 8, 16 and 32 kHz with a 5ms plateau and 1ms rise/fall time at a rate of 5/s. The stimulus intensity ranged from 10 dB sound pressure level (SPL) to 90 dB SPL, with 10 dB increment. ABR threshold was defined as the lowest intensity capable of evoking a reproducible and visually detectable response of Wave II/III complex. Threshold shift represents the difference in threshold recorded after treatment (post-treatment ABR) compared to those recorded on the same animal prior to treatment (pre-treatment ABR). Wave I amplitudes and latencies of ABR by 32KHz at 60, 70,80 and 90 dB SPL were compared between groups.

### Trans-tympanic administration of SB225002 or *CXCR2* siRNA

Rats were administrated trans-tympanically with single dose SB225002 (50 µl of 1.4 nmoles) or vehicle. Also, siCXCR2 (50 µl containing 0.9 µg) or a scramble was delivered *via* tympanic route according to Sheehan et al. (27). Briefly, rats were anesthetized with a mixture of ketamine/xylazine (as above). 50 µl aliquots of vehicle, and SB225002 or siCXCR2 were delivered to inner ear through middle anterior-inferior region on tympanic membrane using 28-30 gauge needle. The administration was monitored using a Zeiss operating microscope. siRNA or scrambled sequence were suspended in nuclease free water and sequentially applied to both ears, with 30 min waiting between each injection.The control animals were injected with scramble or vehicle.

### siRNA sequences

The rat *CXCR2* siRNA was purchased from Ambion by Life Technology (Carlsbad, CA, USA) (#siRNA ID 198228), which includes a mix of two siRNA sequences against *CXCR2*, the sequences of these mixture were 5’ GCGAACCUAGAUAUCAACAtt 3’ and 5’UGUUGAUAUCUAGGUUCGCtg 3’. A non-targeting sequence siRNA for target-specific knockdown (scramble) was purchased from Dharmacon (Lafayette, CO).

### Cochlear whole mount preparation

Cochleae were isolated and perfused with 4% paraformaldehyde through the round and oval windows and kept overnight at 4°C in the same solution. Cochleae were decalcified in 120 mM EDTA (changed daily) for 3 weeks at room temperature, with constant stirring and followed by microdissection to isolate different turns for immunolabeling.

### Hair cells and ribbon synapse count

Cochlear whole-mounts apical, middle and basal turns were imaged using a Zeiss confocal microscope. Microscopic settings were constant for all samples for OHCs and ribbon synapse counting. Samples were labeled with anti-bodies against myosin VIIa, CtBP2 and GluR2 for hair cells staining, presynaptic ribbon and postsynaptic glutamate receptor, respectively. DAPI was used for nuclear staining. Missing OHCs were counted manually for each cochlear turn, with 20X magnification, and presented as percent loss of total number of OHCs. Ribbon synapses from IHCs were imaged using 60X magnification to include at least 15 IHCs per image. Functional (paired) ribbon synapse were defined as those possessing both CtBP2 + GluR2 immunolabeling apposing to each other, whereas orphan (unpaired) synapses were defined as either CtBP2 or GluR2 puncta. The ribbon synapse counting was performed manually from three random microscopic fields for each turn and presented as the number of synapses per IHC.

### RNA isolation and real-time PCR

Cochleae were isolated from treated rats, cleaned from extra tissue and each one was homogenized with 1ml of TRI reagent (Sigma-Aldrich) in DNase/RNase falcon tubes. Chloroform (200 µl) was added to the TRI reagent, the tube was shaken using vortex for 15 sec, and centrifuged at 1200 rpm for 15 min. The aqueous layer (RNA layer) was transferred to another tube. Ice-cold isopropanol (500 µl) was added and mixed by inverting to extract RNA, followed by centrifugation at 12,000 rpm for 10 min. The supernatant was removed carefully and the pellet was washed once with 100% ice-cold ethanol and twice with 75% ethanol in diethyl pyrocarbonate (DEPC)-treated H_2_O. The sample was then centrifuged again at 1,200 rpm for 15 min, the ethanol was removed, and the tube was air dried for 5-10 min. The RNA pellet was suspended in nuclease-free water and the purity of RNA was measured using Nanodrop^®^ ND1000 spectrophotometer (Thermo Fisher, Wilmington DE).

Total RNA (1 µg) was reverse transcribed to cDNA using cDNA Synthesis Kit (Bio-rad, Hercules, CA). The reaction mixture was set up as follow, 1µg of total RNA, 4 µl of iScript reaction mix, 1µl of reverse transcriptase and nuclease free water to bring the volume to 20 µl. The reaction mix was incubated at 25°C for 5 min (priming), then 42°C for 30 min (reverse transcription reaction), followed by 85°C for 5 min (reverse transcription inhibition). The cDNA reaction mix was used for gene expression quantification by StepOne Plus^™^ real time PCR system (Applied Biosystems, Foster City CA). The real-time PCR procedure was set up as follow, 2 µl cDNA (20 ng), 0.4 µl of each primer (200 nM), 10 µl SYBR green master mix (Thermofisher Scientific) and nuclease-free water to bring the volume to 20 µl. The cycling reaction parameters were 95°C for 20 sec (hold), followed by 40 cycles at 95°C for 3 sec (denature) and 60°C for 30 sec (anneal/extend). Gene specific primer pairs were used for reactions and mRNA levels were normalized to the levels of *GAPDH*. The primer sets were purchased from Integrated DNA Technology (Saint Louis, MI) and include the following –

Rodent Nox3 (sense): 5’-ACCAGGCAATTCACATAGCT-3’,
(antisense): 5’CCACAGAAG AACACGCCAA-3’
Rat iNOS:(sense):5’-AAGTACGAGTGGTTCCAGA-3’,
(antisense): 5’-GCACAGCTGCATTGATCTCG-3’
Rat CXCR2 (sense): 5’-CCCTGCCCATCTTCATTCTT-3’,
(antisense): 5’-CACCCTCCACTTGGATGT ATTAT-3’
Rat CXCR1 (sense): 5’-CCTACAATCTGGTTCTGCTCTC-3’,
(antisense): 5’-AG CCCAGGATCTCGGTAATA-3’
Rat CXCL1 (sense): 5’-ATCCCTCAAAGTTCAGTGT-3’
(antisense): 5’-ACGGTTGAGGTAGTCTGA-3’
Rat IL-6 (sense): 5’-ATGAAGTTTCTCTCCGCA-3’,
(antisense): 5’-TATATACTGGTCTGTTGTGG-3’
Rat IL-10 (sense):5’-GTGAAGACTTTCTTTCAAA-3’
(antisense): 5’-TGATCAAGATGTCAAACTC-3’
Rodent STAT1 (sense): 5’-CATGGAAATCAGACAGTACCT-3’,
(antisense) 5’-TCTGTACGGGATCTTCTTGGA-3
Rat STAT3 (Sense):5’-CAGCCAAACTCCCAGATCAT-3’,
(antisense): 5-ACCCAGATTGCCCAAAGATAG-3’
Rodent TNFα (sense):5’-CAGACCCTCACACTCAGATCA-3’,
(antisense): 5’-TGAAGAGAACCTGGGAGTAGA-3’
Rat COX2 (sense): 5’-TCATCGGTGGAGAGGTGTAT-3’,
(antisense): 5’-CTCAGGATGCTCCTGTTTGAG-3’
Rodent GAPDH (sense): 5’-ATGGTGAAGGTCGGTGTGAAC-3’,
(antisense): 5’-TGTAGTTGAGGTCAATGAAGG-3’

### Cell cultures

Immortalized OC cells derived from mouse, UB/OC-1 cells were provided by Dr. Mathew Holly (The University of Sheffield, UK) and cultured in RPMI-1640 media (Hyclone) supplemented with 10% fetal clone^®^ II serum (Hyclone), pencillin-streptomycin (Invitrogen) and normocin (InvivoGen). These cells were grown at 33°C in humidified incubator with 10% CO_2_. Cells were cultured twice a week and all experiments were performed using sub-confluent monolayer of cells.

### Immunohistochemistry

Immunolabeling whole mount sections were initiated by ice-cold 100% methanol for 10 min at -20°C. Sections were then blocked using blocking buffer (10% normal horse or goat serum, 1% BSA and 1% Triton X100) for 2 h at room temperature. The sections were then incubated with primary antibodies diluted with antibody dilution buffer (myosin VIIa 1:200, CtBP2 1:500, GluR2 1:200) overnight at 4°C. On the next day, sections were washed thrice with 1X PBS and incubated with secondary antibodies (1:1000) for 2.5 h. Sections were then counterstained with Hoechst (DAPI) (1:2000) at room temperature for 20 min and mounted with Prolong^®^ Diamond Antifade Mountant (Invitrogen). All the sections were imaged using the same laser illumination settings for all the groups (n≥4 cochleae/group).

Immunohistochemistry for mid-modiolar sections was performed using Immunoperoxidase Secondary Detection System (Millipore USA) for the detection of CXCL1, CD45, CD68 and IBA1. The tissue sections were incubated with 3% H_2_O_2_ for 10 min at room temperature to block the endogenous peroxidase activity. The sections were then washed twice with 1X PBS and incubated in blocking buffer for 1 h at room temperature. The tissue sections were then incubated with 100 µl of the respective primary antibodies (dilutions: 1:50 for CD45, 1:100 for CD68, 1:200 for CXCL1 and 1:200 for IBA1) overnight at 4°C. On the next day, the sections were washed twice with 1X PBS and incubated with biotinylated secondary antibody for 30 min at room temperature. To identify the peroxidase activity, the sections were first incubated with streptavidin HRP for 10 min and then washed twice with 1X PBS. Sections were then incubated with chromogen reagent until the desired stain intensity develops. The sections were then washed in deionized water and counterstained with hematoxylin. The tissue sections were imaged using an Olympus light microscope (Olympus imaging America Inc), using Olympus DP controller software. Semi-quantitative measurement was performed using Image J software version/Fiji Briefly, the intensity was acquired by selecting the region of interest (ROI) for 3 samples per group. Next, the region closes to ROI that had no DAB staining was selected to obtain the background. This value was deducted from the ROI value to compute the intensity of DAB. The results were then plotted as percentage of control where control was considered as 100% (28).

### Measurement proinflammatory CXCL1 levels

For measurement of cytokines and chemokines in cochlea, snap-frozen cochlea were transferred to liquid nitrogen cooled stainless steel tubes containing three 3.2 mm chrome steel beads. A room temperature silicone stopper was inserted into the tube and the stoppered microvial was quickly transferred to the mini-beadbeater-16 (BioSpec Products, Inc., Bartlesville, OK) and bead beat at maximum shaking speed for 15 sec. The vial with powdered tissue was transferred to dry ice to prevent thawing of the tissue and 500 µl of ice-cold sterile phosphate buffered saline containing Complete Protease Inhibitor Cocktail (Roche, Camarillo, CA) was added and the samples were bead beat two cycles x 1 minute with cooling on ice for 5 min between cycles. Homogenates were centrifuged at 13,700 x g at 4°C for 10 min. An aliquot of supernatant was removed and assayed for protein concentration using the BCA protein assay (Pierce Scientific, Rockford, IL). The remaining supernatant was stored in aliquots at -80 C until analysis. A panel of cytokines and chemokines were measured using a multiplex bead-based assay as described by the manufacturer (Bio-Plex Pro kit, Bio-Rad, Hercules, California). Cytokine concentrations that were below the assay limits of detection were assigned the minimal detectable concentration for purposes of statistical analysis.

### Statistics

Data are presented as mean ± standard error of mean (SEM). Statistical significance of differences among groups were tested using t-test, one-way or two-way analysis of variance (ANOVA) depending upon experiments, followed by Bonferroni’s multiple comparison’s test using GraphPad Prism version 6.07 for Windows. P value <0.05 was considered significant.

## Results

### Cisplatin increased the levels of immune cell markers in the cochlea

**Figure 1:**
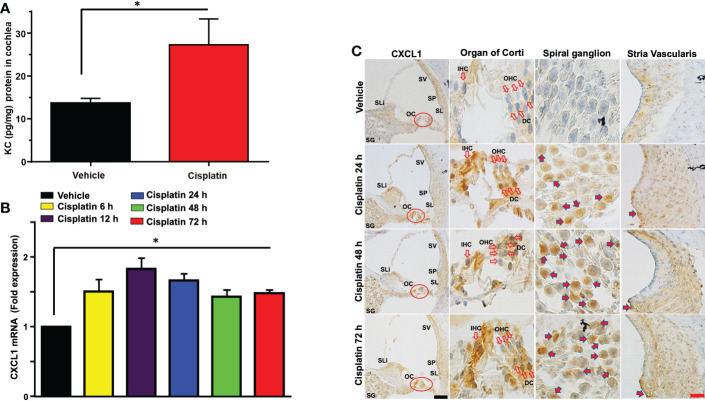
Cisplatin increases the levels and expression of CXCL1 in the cochlea. Male Wistar rats were treated with vehicle or cisplatin (11mg/kg) and sacrificed **(A)** 1 day later and cochleae extracted and processed for CXCL1 using ELISA. **(B)** Quantification of CXCL1 mRNA in the cochlea show significant elevations by 6 h and which remained elevated at least up to 72 h (P<0.0001, F(5,18)=23.20 and Degree of Freedom (D.F.)=23). **(C)** Mid-modiolar sections of cochleae showing CXCL1 immunolabeling over time. Prominent labeling was observed in the organ of Corti (OC) and spiral ganglion (SG). Increased labeling was also observed in the spiral limbus (SLi),spiral ganglion,organ of Corti and auditory nerve. CXCL1 staining was further increased in these regions by 48 h. By 72 h, labeling of CXCL1 in the spiral limbus, auditory nerve, spiral ganglion neurons and organ of Corti was further increased. Some intense labeling of cells is seen in the region of the spiral ligament/spiral prominence. Open arrows indicate OHCs and IHCs. Solid arrows show representative locations where increases in CXCL1 were observed. Asterisk (*) indicates statistical significance from vehicle-treated rats which was determined by analysis of variance (one-way ANOVA). The data is plotted as mean ± SEM (n ≥ 6). Scale bars – black = 50 µm, red = 20 µm.

Male Wistar rats were treated with cisplatin (11 mg/kg) and vehicle (n=6 for each group). Then, the levels of cytokines were determined in cochlear samples 24 h later. Cisplatin treated rats showed significant weight loss over a 3-day period averaging 15-20% body weight. We observed significant increases in several cytokines in the cisplatin treated group compared to vehicle-treated rats assessed at the same time. These include GM-CSF, IL-10, CXCL1, MCP-1, MIP-3a and Rantes. We focused on the neutrophil chemotactic chemokine, CXCL1 which showed significant increases in levels in the cochlea at 24 h (**Figure 1A**). Subsequently, we examined whether these increases were supported by increases in *CXCL1* RNA and assessed the time course of induction in this gene. We observed significant increases in *CXCL1* mRNA by 6 h following cisplatin injections, which remained elevated up to 72 h, the last time point examined (**Figure 1B**). We next examined the localization of CXCL1 in the naïve cochleae and at different times following administration of cisplatin by DAB-based immune-staining. We observed low expression of CXCL1 immunoreactivity, mainly to spiral ganglion and spiral limbus (SLi). Some labeling was observed in the organ of Corti (OC), where immunoreactivity was present in outer (OHCs), inner hair cells (IHCs) and supporting Deiters’ cells (DCs, see red arrows). Cochlear sections from rats treated for 24 h showed increases in staining in the spiral limbus (SLi) and organ of Corti (OC) (**Figure 1C**). Surprisingly, increases in labeling were also observed in non-immune cells such as the SG neurons and OC. By 72 h, labeling of CXCL1 in the SLi, auditory nerve and SG was further increased. Some intense labeling of cells is seen in the region of the SL/spiral prominence(SP) (**Figure 1C**). Quantification of CXCL1 immunolabeling is provided in **Supplementary Figure 1**.

**Figure 2:**
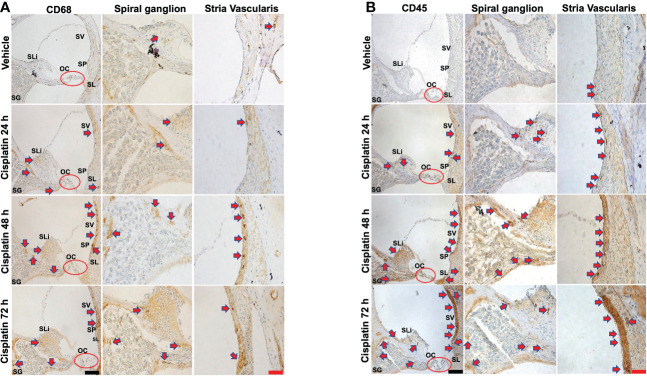
Cisplatin increases the cochlear migration of inflammatory cells. Male Wistar rats administrated with cisplatin (11mg/kg, i.p.) and the rats (n=6 in each group) were sacrificed 24, 48, and 72 h later. Mid-modiolar sections of cochleae were prepared to examine the migration of immune cells based on the expression of CD68 (mainly macrophage) and CD45 (neutrophils and other white cells). **(A)** CD68 labeling was observed in the stria vascularis, spiral ligament and spiral ganglion which was enhanced by cisplatin treatment for 24 h and was progressively increased over a 72h period, especially in the stria vascularis and spiral limbus. **(B)** CD45 labeling showed time-dependent increases starting at 24 h and becoming more intense by 72 h. Labeling was observed in the stria vascularis and spiral ligament, spiral limbus and in regions surrounding the spiral ganglion and auditory nerve. Labeling in the stria vascularis was substantially elevated by 48 and 72 h, especially in the marginal and deeper cell layers. Darker stained areas in the stria vascularis in **(A, B)** appear to be associated with perivascular macrophages. Red arrows indicate regions showing increases in CD68 and CD45 immunolabeling. Scale bars – black = 50 µm, red = 20 µm.

CD68 is a lysosomal, oxidized low density lipoprotein (LDL), rat macrophage and microglia marker, presented in lysosomal associated membrane protein (a family of glycoprotein). CD68 antibody was further used to label immune cell population, mainly macrophages (29). Cisplatin treatment for 24 h labeling was observed in the SV SLi and regions surrounding the SG. These areas showed more intense labeling at 48 and 72h, especially the SLi and the SV. In the SV region, darker staining of CD68 was observed, where the antibody likely stains perivascular macrophages (shown in cross section) (**Figure 2A**). Quantification of CD68 immunolabeling for SG and SV in **Figure 2A** is provided in **Supplementary Figure 2A**.

CD45 (leukocyte common antigen) is a receptor tyrosine phosphatase which is expressed on white blood cells. It is important for the function of neutrophils (30) and essential for T lymphocyte activation *via* the T cell receptor (31) and in integrin-mediated adhesion of macrophages (32). CD45 antibody was used to label the cisplatin-induced accumulation of the immune cell population in the cochlea, as previously described (24, 33–35). Similar to CD68, we observe time-dependent increases in CD45 immunolabeling starting at 24 h and becoming more intense by 72 h (n=6 for each group). Labeling was especially prominent in the SV and SL. Increased labeling was also observed in the SLi and in regions surrounding the SG and auditory nerve. Labeling in the SV was especially intense by 72 h. (**Figure 2B**). Quantification of CD45 immunolabeling in **Figure 2B** is provided in **Supplementary Figure 2B**.

**Figure 3:**
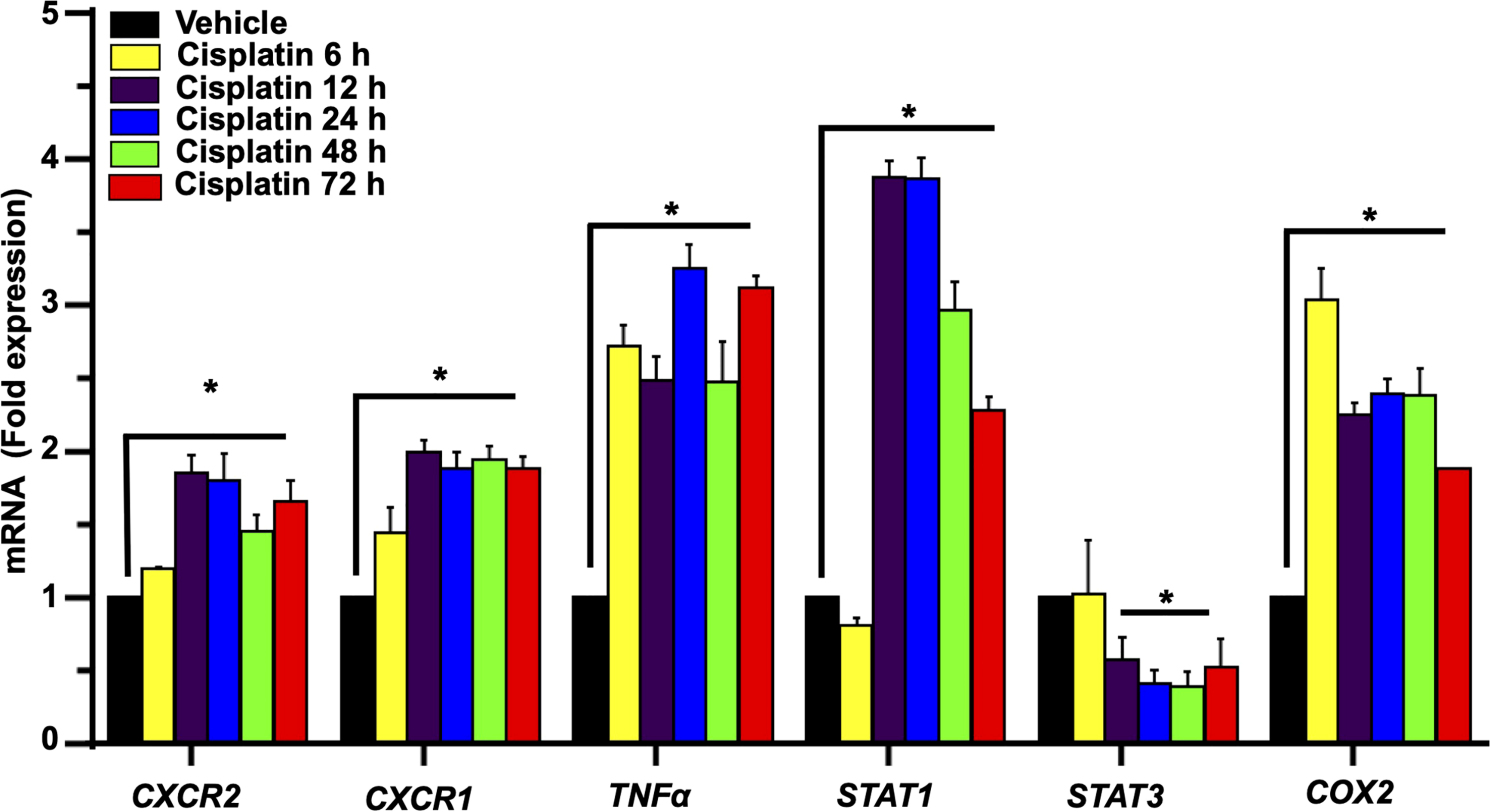
Cisplatin increases the expression of chemokine/chemokine receptors and other inflammatory markers in the cochlea. Male Wistar rats were administered intraperitoneal cisplatin (11mg/kg) and the induction of inflammatory genes were assessed using time course studies for 6, 12, 24, 48, 72 h. The expression of cochlear *CXCR2, CXCR1, CXCL1, TNFα* and *COX2* genes were determined by q-PCR using *GAPDH* as an internal control (housekeeping gene) for normalization. Cisplatin significantly increased *CXCR2, CXCR1, CXCL1, TNFα* and *COX2* expression after 6 h while *STAT1* was increased only after 12 h of treatment (P<0.0003, F(5,25)=7.273 D.F. = 35). The level of *STAT3* expression showed no change after 6 h of cisplatin treatment, but was significantly decreased by 12 h. Asterisk (*) indicates statistical significance from vehicle-treated rats which was determined by analysis of variance (one-way ANOVA). The data is plotted as mean ± SEM (n ≥ 6).

We next examined the expression of several relevant inflammatory genes in a similar time period (as above) in vehicle- and cisplatin-treated Wistar rats. The expression of these mRNAs was low in the vehicle-treated rats but increased substantially following exposure to cisplatin, with different temporal profiles over 3 days. Increases in *CXCR2* and *CXCR1* (receptor targets for CXCL1) were significantly elevated by 12 h and maintained at these levels up to 72 h. The levels of *TNFα* and *COX2* showed increases by 6 h and these levels were maintained up to 72 h. The levels of the transcription factors, *STAT1* and *STAT3* (which are associated with cisplatin-induced hearing loss) showed differential regulation, with the levels of *STAT1* showing peak increases in 12 h, which diminished over 72 h. In contrast, the levels of *STAT3* were suppressed by 12 h and remained reduced over 72 h (**Figure 3**).

**Figure 4:**
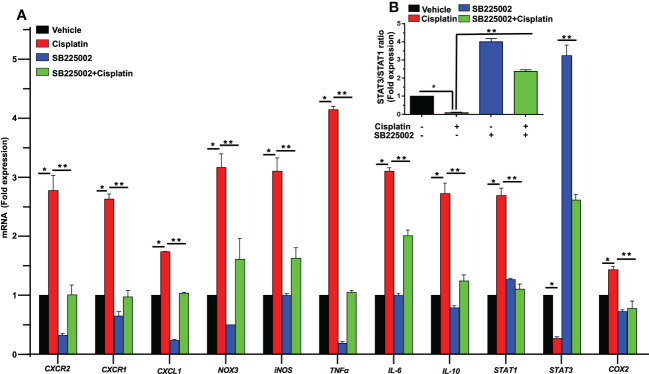
SB225002 suppresses cisplatin modulation of pro-inflammatory genes. **(A)** Cisplatin induced inflammatory genes were assessed 72 h following cisplatin treatment. The expression of cochlear *CXCR2, CXCR1, CXCL1, NOX3, iNOS, TNFα, IL-6, IL-10, STAT1* and *COX2* genes were significantly increased by cisplatin. In rats treated with SB225002 (1.4 nmoles/ear) the response to cisplatin was attenuated (P>0.0008, F(3,40)=6.8 and D.F. = 43) **(B)** The ratios of *STAT3:STAT1*, derived from **(A)**, were suppressed by cisplatin, normalized in the SB225002 + cisplatin group but significantly enhanced by SB225002 alone. Asterisk, (*) indicates significant difference from vehicle group while (**) indicate statistically significant difference from cisplatin-treated group. Statistical significance from vehicle-treated rats which was determined by analysis of variance (one-way ANOVA).

To determine a functional role of CXCL1 and its receptor targets in the cochlea, we used the drug SB225002 to block the CXCR2 target of this chemokine (36). In **Figure 4A**, we show that cisplatin treatment produced significant increases in the expression of *CXCR2*, *CXCR1* and *CXCL1* by 2.8 ± 0.3, 2.6 ± 0.1 and 1.7 ± 0.1-fold, respectively, by 72 h. Other inflammatory genes such as *IL-6*, *IL-10*, and *STAT1* were also increased by 3.1 ± 0.1, 2.7 ± 0.2, and 2.6 ± 0.1-fold, respectively (**Figure 4A**). The expression of these genes in rats pretreated (trans-tympanically) with SB225002 (1.4 nmoles/ear) followed by cisplatin were significantly reduced for *CXCR2*, *CXCR1*, *CXCL1, IL-6*, *IL-10*, and *STAT1* to 1.0 ± 0.2, 1.0 ± 0.1, 1.0 ± 0.1, 2.0 ± 0.1, 1.2 ± 0.1 and 1.1 ± 0.1-fold, respectively (**Figure 4A**). STAT1-regulated genes such as *NOX3*, *iNOS*, *TNFα* and *COX2* were also up regulated by cisplatin by 3.2 ± 0.2, 3.1 ± 0.2, 4.1 ± 0.1 and 1.4 ± 0.1-fold, respectively. Inhibition of CXCR2 by SB225002 reduced the expression for these genes to 1.6 ± 0.4, 1.6 ± 0.2, 1.0 ± 0.1 and 0.8 ± 0.1 respectively. We examined the ratio of *STAT3*/*STAT1*, a marker of cell stress (as above). Interestingly, we observed that while cisplatin decreased this ratio (by increasing *STAT1* and decreasing *STAT3*), trans-tympanic administration of SB225002, not only restored this ratio but increased it to ~2-fold (**Figure 4B**).

**Figure 5:**
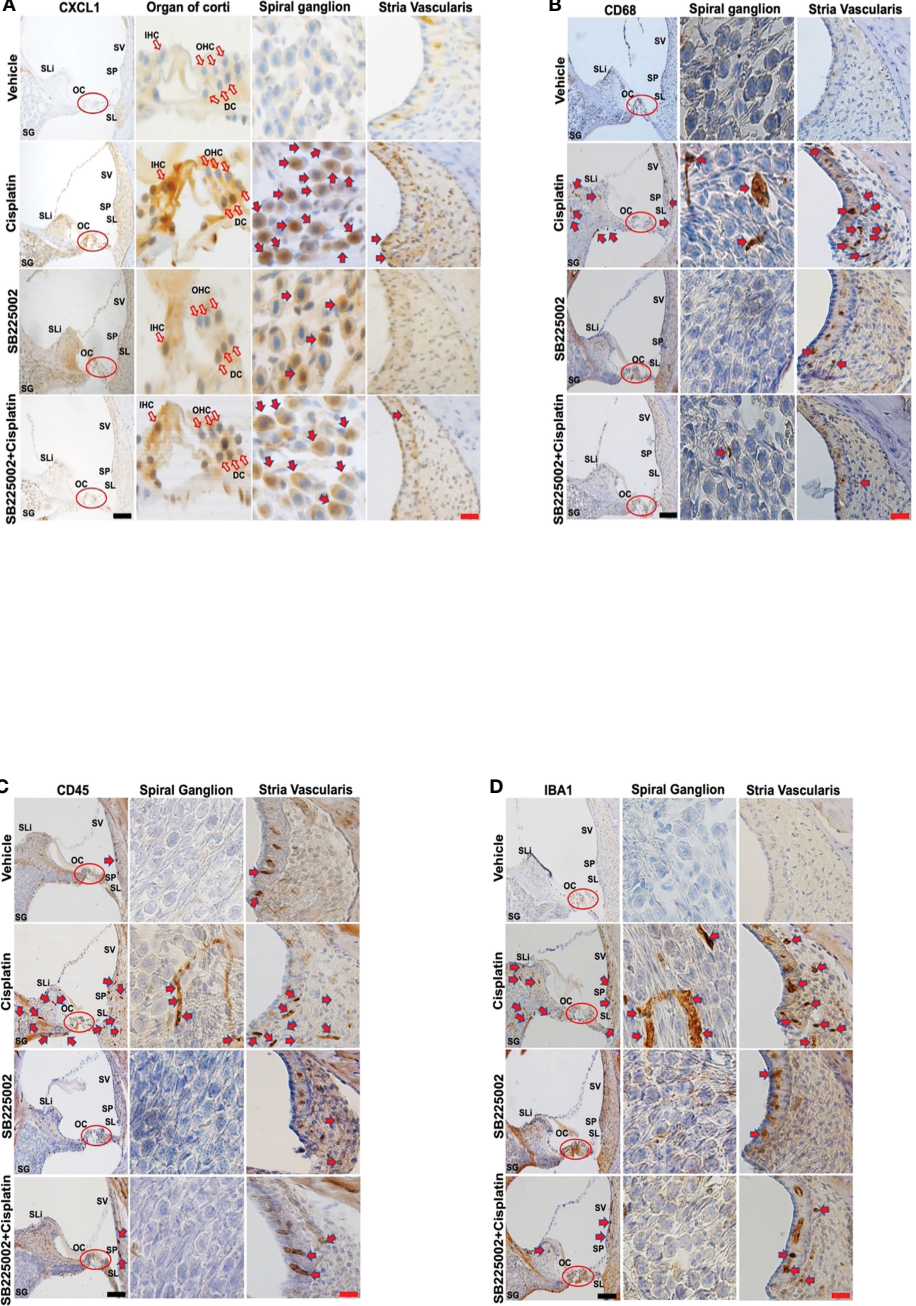
Trans-tympanic administration of SB22500 reduced inflammatory cell markers in the cochlea induced by cisplatin. **(A)** Wistar rats were administered vehicle or SB225002 in both ears, followed by cisplatin (11 mg/kg) 30 min later. Rats were sacrificed 72 h later and cochleae were processed for mid-modiolar sectioning. The level of CXCL1, CD68, CD45 and IBA1 was detected by diaminobenzidine tetrahydrochloride (DAB) staining while cell nuclei were stained with hematoxylin stain. DAB staining revealed in high levels of CXCL1 (dark-brown labeling) in OC, SG, SLi and in cisplatin-treated rat cochleae over that obtained in vehicle-treated controls. Blockade of CXCR2 by SB225002 attenuated the increases in CXCL1 immunoreactivity compared to the rats treated with vehicle plus cisplatin. **(B)** Immunolabeling for CD68 showed increased number of dark brown stained cells in the different region of SVA, SL and in areas surrounding the SG neurons. Inhibition of CXCR2 by SB225002 reduced the number of cells in the SVA. Dark brown staining in the intermediate layer likely represent staining of perivascular macrophage. **(C, D)**, CD45 and IBA1 immunolabeling were increased in SVA, SL and SLi following cisplatin treatment. Lower levels of immunolabeling were observed in the animals pretreated with SB225002, followed by cisplatin. Figures shown are a representative of similar obtained in six independent animals per treatment group. Open arrows in **(A)** indicate hair cells while solid arrows represent labeling of SG neurons and SV. Solid arrows in panels b-d represent labeling of immune cells in the SG, SV and SL regions of the cochlea. Increased labeling of immune cells in the SV and SL was observed following cisplatin administration. Scale bars – black = 50 µm, red = 20 µm.

We next examined the effect of SB225002 on CXCL1 immunolabeling in the cochlea and migration of immune cells in rats treated with cisplatin. We observed that while cisplatin increased the levels of CXCL1 (as above), pretreatment with SB225002 attenuated these increases in the OC and SG (**Figure 5A**). Quantification of CXCL1 immunolabeling in SG and SV is presented in **Supplementary Figure 3A**. Similarly, we noticed that the observed increase in CD68 immunolabeling in the SV, SG, SL which were suppressed by SB225002 (**Figure 5B**). Increased immunolabeling of other immune cell markers, such as CD45 (**Figure 5C**) and IBA1 (**Figure 5D**) were also suppressed by SB225002. These data suggest that blockade of CXCR2 reduces cochlear inflammatory gene expression, CXCL1 immunolabeling and the entry of CD68 and CD45 immune cells into the cochlea. Quantification of CD68, CD45 and IBA1 immunolabeling for SG and SV in **Figures 5B–D** are provided in **Supplementary Figures 3B–D** respectively.

### Inhibition of CXCR2 protects against cisplatin-induced hearing loss

**Figure 6:**
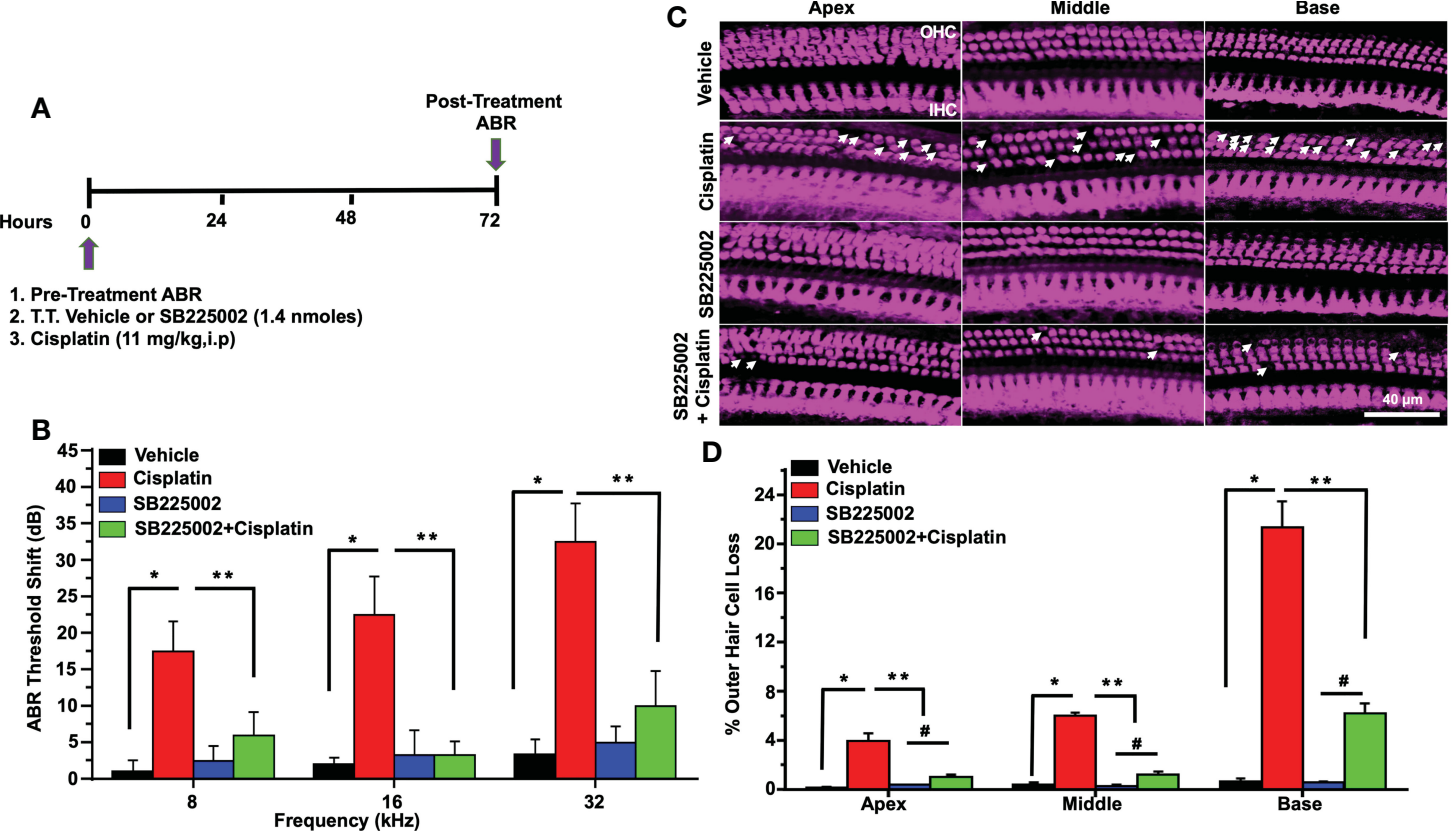
Trans-tympanic delivery of SB225002 attenuated cisplatin-induced hearing loss. **(A)** Figure depicts experimental procedure that describes the dosage and route of administration for SB225002 (1.4 nmoles/ear) and cisplatin (11mg/kg). **(B)** ABR thresholds were recorded in Wistar rats which were then treated trans-tympanically with vehicle or SB225002 in both ears. Rats were then administered with vehicle or cisplatin (11 mg/kg) and ABRs were recorded 72 h later. Cisplatin produced a significant increase in ABR thresholds at 8,16 and 32 KHz frequencies which were attenuated by SB225002 at all frequencies tested. Data represent mean ± SEM of four rats (P<0.0001 between vehicle, cisplatin and SB225002+cisplatin, F(3,62)=19.89 and DF=62). **(C)** Cochleae were isolated, decalcified for 21 days with 120 mM EDTA (daily changed) and used for preparing whole mounts. Basal, middle and apex turns were stained with Myosin VIIa (magenta) to visualize OHCs and IHCs. Representative whole-mount images showed significant OHCs damage (white arrow) by cisplatin, while trans-tympanic pretreatment with SB225002 protected OHCs. Scale bar represent 20 µm. **(D)** Percentage missing OHCs in basal, middle and apex turns of cochlea were significantly decreased by pretreatment with SB225002 compared with cisplatin treated group. No loss of hair cells was observed in the vehicle or SB225002-treated cochleae (P<0.0001 between vehicle cisplatin and SB225002+cisplatin, F(3,12)=71.13 and DF=15) Data indicates mean ± SEM. Asterisks, (*) indicates significant difference from vehicle group, while (**) indicate significant difference form cisplatin group (n=4), (#) Indicate statistically significant difference from the cisplatin-treated group and from vehicle group. Statistical analyses among groups were tested using one-way analysis of variance (ANOVA).

In order to determine whether CXCL1 is implicated in cisplatin-induced hearing loss, we performed functional studies in rats treated with cisplatin, as described above. CXCR2 is a target of CXCL1 (37). Pre-treatment ABRs were obtained from all rats (n=4) which were then treated with trans-tympanic vehicle or SB225002 (1.4 nmoles/ear), followed by intraperitoneal vehicle or cisplatin (11 mg/kg) injection 30 min later. Post-treatment ABRs were measured 72 h following vehicle or cisplatin administration (**Figure 6A**). Vehicle administration alone produced small changes in ABRs, but were comparable to the pre-treatment ABRs. Cisplatin significantly increased ABRs threshold shifts to 17.5 ± 4.1, 22.5 ± 5.3 and 32.5 ± 5.3 dB at 8, 16, and 32 kHz, respectively. Pretreatment with SB225002 significantly attenuated the elevations in ABR threshold shifts at all frequencies tested, with these values being 6.0 ± 3.2, 3.3 ± 1.8 and 10.0 ± 4.8, respectively (**Figure 6B**) The administration of SB225002 alone produced some ABR shifts, but these were below 5 dB at all frequencies tested. Cisplatin-induced hearing loss was associated with the loss of OHCs in the apical, middle and basal turns of the cochlea. Manual counting of OHCs in whole-mount preparations (using myosin VIIa labeling) in cisplatin-treated group showed loss of OHCs in the apical, middle and basal turns averaging 3.9 ± 0.6, 6.0 ± 0.2 and 21.4 ± 2.1%, respectively. SB225002 significantly reduced these losses to 1.0 ± 0.2, 1.3 ± 0.2 and 6.2 ± 0.9% in the apical, middle and basal turns, respectively. Administration of SB225002 alone had no significant effect on OHCs (**Figures 6C, D**). As expected, we did not observe any loss of IHCs with cisplatin or with cisplatin plus SB225002 within the 3-day time period. These results suggest that blocking CXCR2 by SB225002 using the trans-tympanic route can effectively reduce cisplatin ototoxicity in the rat model, assessed at 72 h post-treatment.

### CXCR2 antagonist reduces cisplatin-induced cochlear synaptopathy in rats

**Figure 7:**
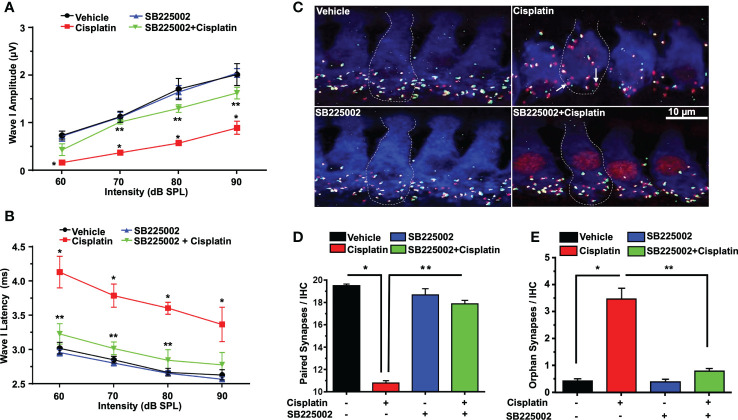
SB225002 reduces cisplatin-induced loss of synaptic function and ribbon synapses. ABR Wave 1 amplitudes **(A)** and latencies **(B)** were collected from all treatment groups at 60, 70, 80, 90, dB SPL (32 KHz). **(A)** Cisplatin significantly decreases supra-threshold amplitudes while SB225002 (1.4 nmoles/ear) protected against cisplatin effects (P<0.0001 between vehicle cisplatin and SB225002+cisplatin, F(3,86)=74.39 and DF=86). Data represent mean ± SEM of four rats. Statistical analyses were performed using two-way analysis of variance (ANOVA). **(B)** Cisplatin significantly increased supra-threshold Wave 1 latencies which were reduced by SB225002 (P<0.0001 between vehicle cisplatin and SB225002+cisplatin, F(3,88)=43.43 and DF= 88). Statistical analyses were performed using two-way analysis of variance (ANOVA). **(C)** Male Wistar rats were pretreated with vehicle or SB225002 30 min later, cochleae were collected and subjected to whole-mount dissection to isolate basal, middle and apex turns. turns sections were stained with hair cell marker, myosin VIIa (blue), CtBP2 pre-synaptic marker (red) and GluR2 post-synaptic marker (green). Representative whole-mount images from turns showed that cisplatin reduced the number of ribbon synapse per IHC which was blunted by trans-tympanic SB225002. Orphan synapses represent staining by either GluR2 or CtBP2 alone which were not paired (indicated by white arrows). **(D)** The number of synaptic ribbons per IHC in the basal turn was reduced by cisplatin, but this response was attenuated by SB225002 (P<0.0001 between vehicle cisplatin and SB225002+cisplatin, F(3,28)=138 and DF=31). **(E)** Cisplatin significantly induce the number of orphan synapses per IHC which was protected by SB225002 (P<0.0001 between vehicle cisplatin and SB225002+cisplatin, F(3,28)=47.8 and D.F equals 31). Asterisks, (*) indicates significant difference from vehicle group, while (**) indicate significant difference form cisplatin group. Statistical analyses among groups were tested using one-way analysis of variance (ANOVA). Data indicates mean ± SEM.

Acoustic trauma, aging, and ototoxic drugs contribute to loss of ribbon synapses and SG (38–42). Cochlear synapse pathology is defined as loss of synapses between type I SG afferents and IHCs which results in deficiency in signal coding, atrophy and decreases in supra-threshold ABR wave I amplitudes and increased latencies (21). Cisplatin significantly decreased wave I amplitudes at 60, 70, 80, and 90 dB SPL intensities to 0.16 ± 0.09, 0.36 ± 0.03, 0.57 ± 0.04 and 0.89 ± 0.1 µV, respectively, compared to the respective values in the vehicle-treated group which were 0.73 ± 0.09, 1.12 ± 0.11, 1.71 ± 0.22 and 2.01 ± 0.18 µV. Trans-tympanic injection of SB225002 decreased the extent of the reductions in wave I amplitudes at intensities 70, 80, and 90 dB SPL by cisplatin to 1.01 ± 0.04, 1.3 ± 0.08 and 1.62 ± 0.12 µV, respectively (**Figure 7A**).

Wave I latencies were analyzed for 60, 70, 80, and 90 dB SPL sound intensities at 32 KHz. Cisplatin significantly increased wave I latencies at 60, 70, 80, and 90 dB SPL intensities to 4.12 ± 0.22, 3.78 ± 0.16, 3.6 ± 0.08 and 3.36 ± 0.03 ms, respectively, compared to vehicle-treated controls which were 3.02 ± 0.08, 2.85 ± 0.04, 2.66 ± 0.05 and 2.63 ± 0.07 respectively. Pretreatment with SB225002 diminish cisplatin-induced prolongation of latencies which were 3.20 ± 0.14, 3.01 ± 0.09 and 2.84 ± 0.15 ms, respectively, at 60, 70 and 80 dB. No significant change was observed at 90 dB SPL, even though a similar trend was observed (p=0.11) (**Figure 7B**).

Loss of ribbon synapse was reported following noise exposure (43), cisplatin administration (44–46), aminoglycoside (47) and aging (41). We examined the pre-synaptic ribbons, which are labeled by an antibody against C terminal protein 2 (CtBP2), and the post-synaptic glutamate receptor, labeled by antibody for GluR2. The loss of synapse were validated by immunolabeling of whole-mount sections with antibodies against these synaptic proteins. IHCs were labeled with antibodies against myosin VIIa (blue). We observed that the average number of paired synapses, identified by co-labeling of both CtBP2 (red fluorescence) and GluR2 (green fluorescence) per IHC in the basal turn of vehicle was 19.5 ± 0.2, which was significantly decreased to 10.7 ± 0.2 following cisplatin treatment. Paired synapses were detected as yellow fluorescence (combination of red plus yellow). Trans-tympanic injection of SB225002 significantly rescued the loss of paired synapses observed with cisplatin to 17.9 ± 0.5 (**Figures 7C, D**). The number of orphan synapse (CtBP2 or GluR2 staining not paired with each other) was increased from 0.8 ± 0.1 per IHC in vehicle treated rats to 2.0 ± 0.2 by cisplatin. Pretreatment with SB225002 protected against the increases in orphan synapses by cisplatin, which averaged 0.9 ± 0.1 (**Figures 7C, E**). Overall, these findings indicate that SB225002 can reduce cisplatin-mediated cochlear synaptopathy.

No loss of OHC was observed following 24 h of cisplatin administration (**Supplementary Figure 4**) and this correlated with the lack of ABR shifts in the cisplatin-treated rats (n=4) measured at this time (data not shown). However, we observed significant loss in paired ribbon synapses at this time which averaged ~20% and a significant increase in orphan synapses. These changes were reduced by pretreatment with SB225002. These data implicate CXCR2 activation in cisplatin-induced loss of ribbon synapses and indicate that cisplatin-induced loss of ribbon synapses precedes ABR shifts and loss of OHCs in the cochlea.

### Knockdown of *CXCR2* in the cochlea reduces cisplatin-induced hearing loss, synaptopathy and pro-inflammatory genes

**Figure 8:**
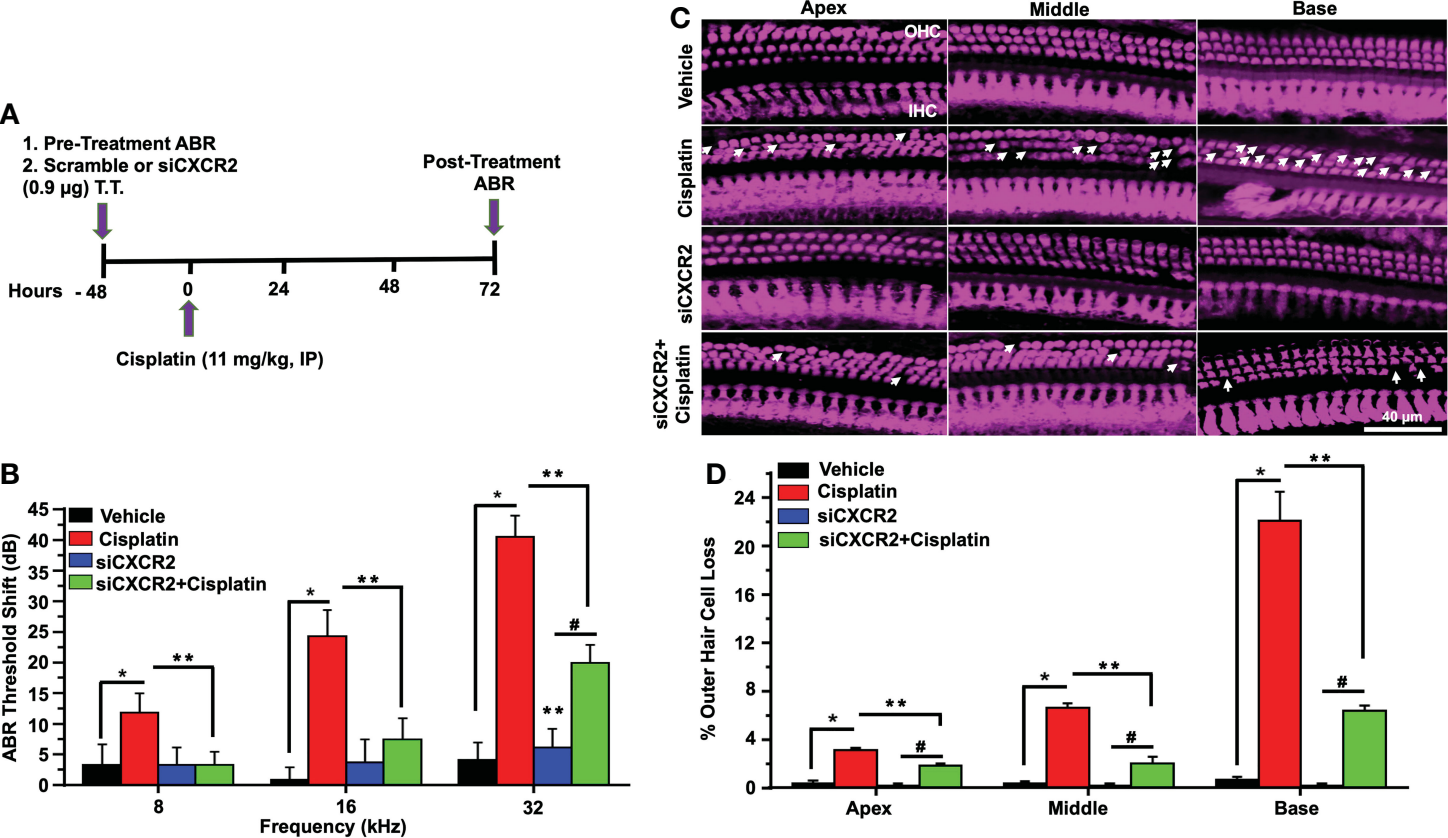
CXCR2 knockdown attenuated cisplatin-induced hearing loss. **(A)** A diagram represents experimental procedure that describes the dosage and route of administration for CXCR2 siRNA (siCXCR2) and cisplatin (11mg/kg). **(B)** Pretreatment ABR thresholds were recorded in rats, which were then injected by the trans-tympanic route with vehicle or siCXCR2 (0.9 µg) in both ears. Rats were then administered cisplatin 48 h later by intraperitoneal injections. Post-treatment ABRs were recorded 72 h following cisplatin administration. Cisplatin increased ABR thresholds for all frequencies while knockdown of CXCR2 attenuated this response (P<0.0001 between vehicle cisplatin and siCXCR2+cisplatin, F(10,132)=24.29 and DF=132). **(C)** Cochleae were collected and prepared, as described in Methods, and whole-mount sections were dissection to isolate the three different turns of the cochlea. Sections were stained for myosin VIIa (magenta). Representative whole-mount images show significant OHCs damage (white arrow) by cisplatin, while trans-tympanic siRNA (0.9 µg) protects OHCs damaging caused by cisplatin. Scale bar represent 20 µm. **(D)** Percentage of missing OHCs in basal, middle and apex turns of cochlea which was significantly attenuated by siCXCR2 (P<0.0001 between vehicle cisplatin and siCXCR2+cisplatin, F(3,42)=103.5 and DF=45) Data are presented as the mean ± SEM. Asterisks (*) indicates significant difference from vehicle group, while (**) indicate significant difference form cisplatin. (#) Indicate statistically significant difference from the cisplatin-treated group and from vehicle group (n=4). Statistical analyses among groups were tested using one-way analysis of variance (ANOVA).

To better establish a role of CXCR2 in mediating cisplatin-induced hearing loss, we examined whether knockdown of *CXCR2* mRNA protects against cisplatin-induced hearing loss. CXCR2 is a prime target for knockdown as it is activated by CXCL1 and macrophage inflammatory protein-2, cytokines which promote neutrophil recruitment to sites of inflammation (48). Rats were administered scramble siRNA or siRNA for *CXCR2* gene (siCXCR2) *via* the trans-tympanic route, followed by intraperitoneal vehicle or cisplatin (11mg/kg) 48 h later (**Figure 8A**). siCXCR2 (0.9 µg/ear) produced ~70% knockdown of *CXCR2* mRNA when compared animals injected with scramble siRNA. Post-treatment ABRs were assessed 72 h following vehicle/cisplatin administration (**Figure 8B**). ABRs recorded from animals administered siCXCR2 alone showed slight changes in thresholds when compared to the pre-treatment values. Cisplatin at all three frequencies increased ABR threshold shifts to 11.8 ± 3.1, 24.3 ± 4.3 and 40.6 ± 3.5 dB at 8, 16, and 32 KHz, respectively. Knockdown of *CXCR2* decreased the respective threshold shifts significantly to 3.3 ± 2.1, 7.5 ± 3.5 and 20.0 ± 3.0 (**Figure 8B**).

Manual counting of OHCs in whole-mount preparations of cisplatin-treated mice indicated 3.1 ± 0.2, 6.7 ± 0.3 and 22.2 ± 2.3% loss of OHCs from apical, middle and basal turns, respectively, while knockdown of *CXCR2* gene significantly reduced this effect of cisplatin to 1.9 ± 0.2, 2.1 ± 0.5 and 6.4 ± 0.4% loss of OHCs from these respective regions, without altering IHC number. Similar to inhibition of CXCR2, these results suggested that knockdown of *CXCR2* can also reduce cisplatin ototoxicity (**Figures 8C, D**).

**Figure 9:**
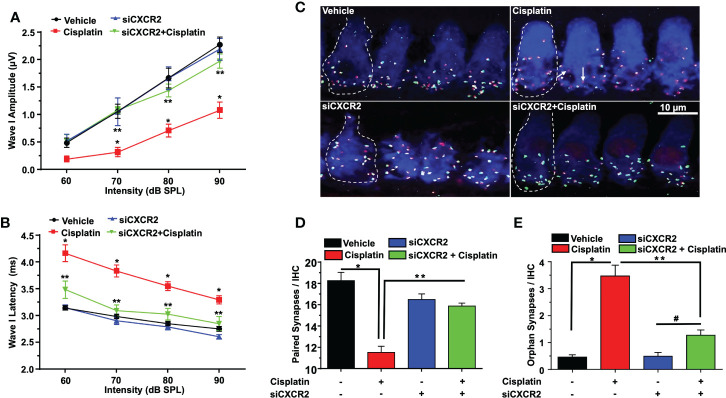
CXCR2 knockdown attenuates cisplatin-induced loss of ribbon synapses. ABR Wave I amplitude **(A)** and latency **(B)** for all treatment groups at 60, 70, 80, 90, dB SPL (32 KHz) were collected from ABR wave forms. **(A)** Cisplatin significantly decreases supra-threshold Wave I amplitudes for 70, 80 and 90 dB SPL intensities but not at 60 dB SPL. Knockdown of *CXCR2* significantly protects against cisplatin the cisplatin response and increased Wave I amplitudes (P<0.0001 between vehicle cisplatin and siCXCR2+cisplatin, F(3,104)=41.1 and DF=104). Data represent mean ± SEM of four rats. Statistical analyses were performed using two-way analysis of variance (ANOVA). **(B)** Cisplatin-induced delay in Wave I latencies was reduced by knockdown of CXCR2 (P<0.0021 between vehicle cisplatin and siCXCR2+cisplatin, F(3,120)=120 and DF=120) Statistical analyses were performed using two-way analysis of variance (ANOVA). **(C)** Cochlear turns sections were stained with hair cell marker, myosin VIIa (blue), CtBP2 pre-synaptic marker (red) and GluR2 post-synaptic marker (green). Representative whole-mount images from turns showed that cisplatin reduced the number of ribbon synapse per IHC, whereas these effects was abolished by siCXCR2. Orphan synapses were depicted as staining with either GluR2 or CtBP2 alone, but not both (indicated by white arrows). Scale bar represent 10 µm. **(D)** Graph shows the number of synaptic ribbons per IHC which were substantially preserved by siCXCR2 (P<0.0001 between vehicle cisplatin and siCXCR2+cisplatin, F(3,10)=28.35 and DF=13). **(E)** Cisplatin significantly induced orphan synapses per IHC which were reduced by siCXCR2 (P<0.0001 between vehicle cisplatin and siCXCR2+cisplatin, F(3,10)=29.57 and DF=13). Data are presented as the mean ± SEM of four animals. Asterisks, (*) indicates significant difference from vehicle group, while (**) indicate significant difference form cisplatin group. # indicates statistically significant increase from siCXCR2 group.

Wave 1 amplitudes of ABR induced by 32 kHz tone at 70, 80, and 90 dB SPL in the scramble siRNA-treated rats were 0.48 ± 0.08, 1.05 ± 0.12, 1.66 ± 0.17 and 2.27 ± 0.13 µV respectively. Wave 1 amplitudes observed 72 h following cisplatin administration were significantly reduced to 0.31 ± 0.08, 0.71 ± 0.11 and 1.07 ± 0.15, respectively, with no significant effect observed at 60 dB SPL intensity. Knockdown of *CXCR2*, followed by cisplatin, led to significantly increased wave I amplitudes at intensities 70, 80, and 90 dB SPL to 1.08 ± 0.05, 1.44 ± 0.11 and 1.97 ± 0.12, while no significant change was showed at 60 dB SPL (p=0.87) (**Figure 9A**). Wave I latencies measured at 60, 70, 80, and 90 dB SPL intensities at 32 KHz, were 4.2 ± 0.2, 3.8 ± 0.1, 3.6 ± 0.1 and 3.3 ± 0.1 msec, respectively. Knockdown of *CXCR2* significantly abolish cisplatin-induced increases in wave 1 latencies at 60, 70, 80 and 90dB SPL to 3.4 ± 0.2, 3.1 ± 0.1 and 3.0 ± 0.1, 2.8 ± 0.13 msec, respectively (**Figure 9B**).

Manual counts of ribbon synapses show that the average number of paired synapses per IHC in the basal turns of control cochleae were 18.2 ± 0.8 per IHC. Cochleae from cisplatin-treated rats (assessed 3 days following cisplatin administration) showed significantly decreased number of paired synapses (11.0 ± 0.6 per IHC). Knockdown of cochlear *CXCR2* significantly reduce cisplatin-mediated loss of paired synapses which averaged 15.8 ± 0.3 (**Figures 9C, D**). The number of orphan (unpaired) synapse was significantly increased from 0.5 ± 0.1 per IHC in vehicle treated rats to 3.4 ± 0.4 after cisplatin treatment. Pretreatment of rats with siCXCR2 significantly protected against the increase in orphan synapses obtained with cisplatin, with the number of orphan synapses averaging 1.3 ± 0.2 (**Figures 9C, E**).

**Figure 10:**
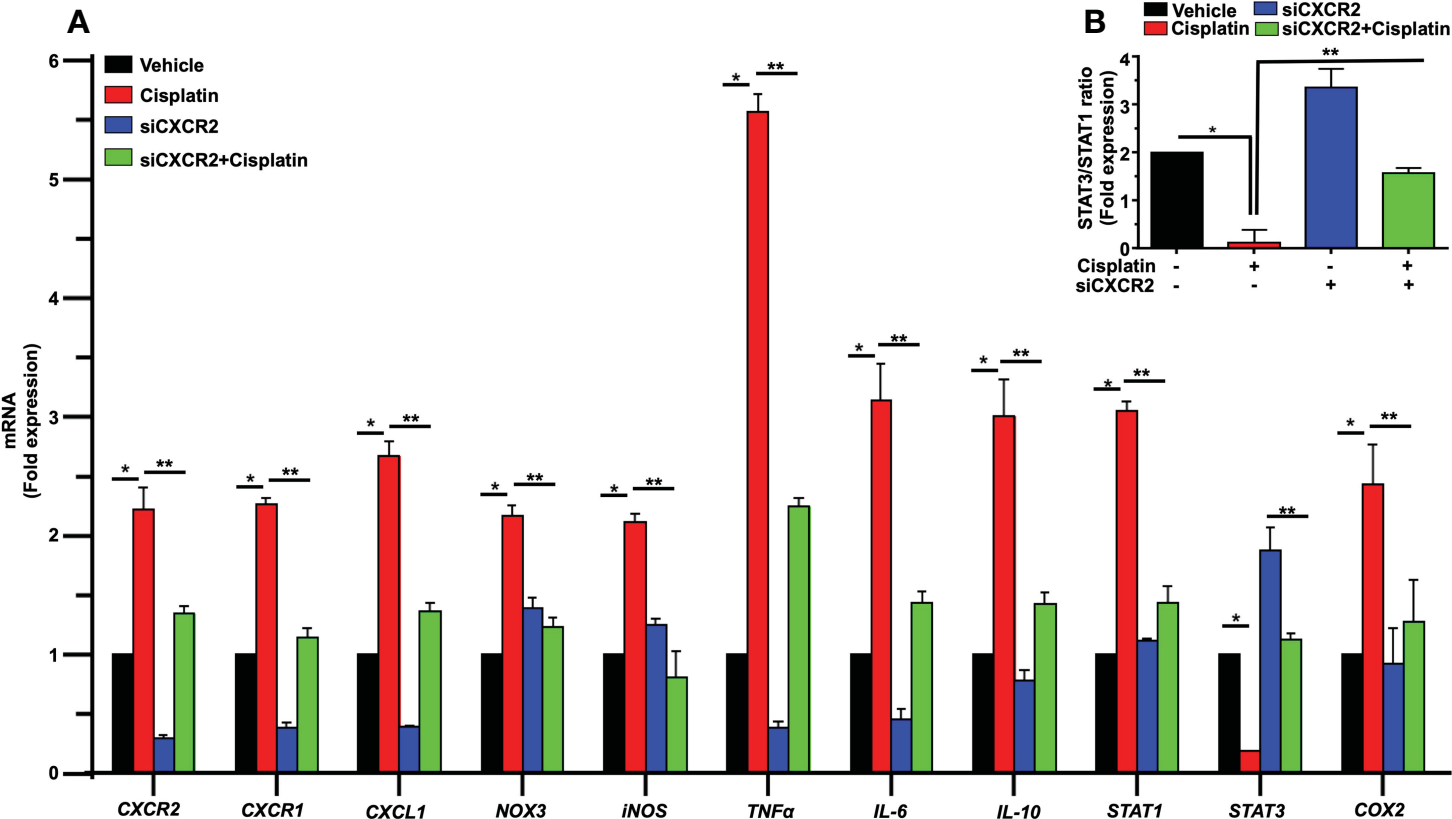
CXCR2 knockdown inhibits cisplatin-mediated expression of inflammatory genes response in rat cochlea. Male Wistar rats were injected with scramble or CXCR2 siRNA (siCXCR2 0.9 µg) by the trans-tympanic route, 48 h later animals followed by cisplatin (11mg/kg). Rats were sacrificed 72 h later, and their cochleae collected for RNA preparation. The expression levels of *CXCR2, CXCR1, CXCL1, NOX3, iNOS, TNFα, IL-6, IL-10, STAT1, STAT3, COX2* genes are presented following normalization with *GAPDH.* Cisplatin induced inflammatory genes response, whereas knockdown of *CXCR2* reduced cisplatin-induced response. The exception is STAT3 which was reduced by cisplatin and recovered in the *CXCR2* knockdown group (P<0.0001 between vehicle cisplatin and siCXCR2+cisplatin, F(30,132)=28.97 and DF=132). **(B)** The ratio of *STAT3:STAT1*, derived from **(A)** was suppressed by cisplatin, normalized in the siCXCR2 + cisplatin group but significantly enhanced by siCXCR2. Data were presented as fold change mean ± SEM (n=4). Asterisks (*) indicate significant difference from vehicle group while (**) indicate significant difference form cisplatin group (n=4). Statistical analyses among groups were tested using one-way analysis of variance (ANOVA).

As observed previously, cisplatin significantly enhanced the expression of cochlear genes linked to inflammation, including *CXCR2*, *CXCR1* and *CXCL1*. The fold changes were 2.2 ± 0.2, 2.3 ± 0.1 and 2.8 ± 0.1 respectively, which were significantly reduced by siCXCR2 to 0.29 ± 0.02, 0.38 ± 0.03 and 0.38 ± 0.01 respectively. In addition, the expression of STAT1-regulated genes, such as *NOX3*, *iNOS*, *TNFα* and *COX2* were upregulated by cisplatin to 2.1 ± 0.1, 2.1 ± 0.1, 5.5 ± 0.2, and 2.4 ± 0.1-fold, respectively, compared to the vehicle-treated animals. These latter genes were significantly reduced in siCXCR2 + cisplatin-treated rats to 1.2 ± 0.1, 0.8 ± 0.2, 2.2 ± 0.1 and 1.2 ± 0.4-fold, respectively (**Figure 10A**). Cisplatin also increased the expression of *IL-6*, *IL-10*, and *STAT1* by 3.1 ± 0.3, 3.0 ± 0.3, and 3.0 ± 0.1-fold, respectively, while the expression level of these genes in siCXCR2 + cisplatin-treated group were significantly reduced to 1.4 ± 0.1, 1.4 ± 0.1 and 1.4 ± 0.1-fold, respectively (**Figure 10A**). In contrast to other genes, the level of *STAT3*, a transcription factor associated with cell survival (49), was significantly reduced by cisplatin and restored in the siCXCR2 + cisplatin group. This overexpression of *STAT3* in the siCXCR2 + cisplatin group is similar to that observed in the SB225002 + cisplatin group (**Figure 10B**) and further support a role of CXCR2 in mediating cisplatin suppression of *STAT3* expression. The expression of *STAT3* was suppressed by cisplatin, while this suppression was reversed and significantly enhanced by trans-tympanic siCXCR2 (0.9µg). Therefore, we assessed the ratios of *STAT3:STAT1* following siCXCR2 administration. (**Figure 10B**) shows that cisplatin reduced the *STAT3:STAT1* ratio, while siCXCR2 blocked this reduction and even enhanced this ratio when added alone. These data support a role of CXCR2 in induction of *STAT1* and in suppressing *STAT3* expression. They further indicate a tonic regulation of *STAT3* by CXCR2.

### Examination of CXCL1-CXCR2 signaling *in vitro*

**Figure 11:**
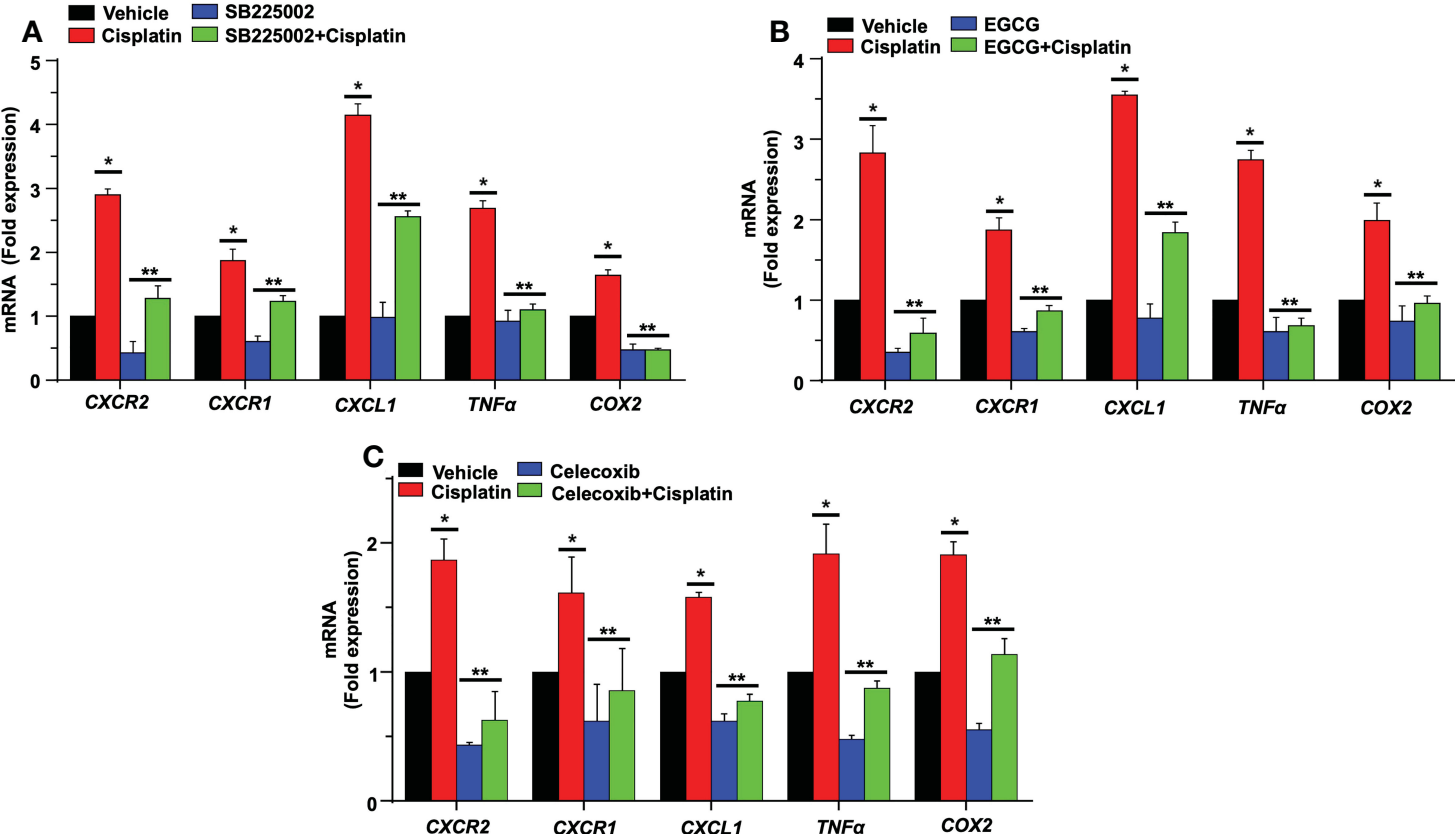
SB225002 and EGCG modulate cisplatin-induced inflammatory gene in UB/OC-1. **(A)** UB/OC-1 cells were pretreated with vehicle or SB225002 (300 nm) for 30 min, followed by cisplatin treatment (2.5 µM) for 24 hand RNA was isolated. The expression of the inflammatory genes *CXCR2, CXCR1, CXCL1, TNFα* and *COX2* were determined by q-PCR using *GAPDH* as an internal control (housekeeping gene) for normalization. Cisplatin increased the expression of these genes while SB225002, an inhibitor of CXCR2, significantly reduced the induction of these genes (P<0.0009, F(3,16)=9.195 and DF=19). Data are presented as the mean fold change ± SEM (n=4). Asterisk, (*) indicates significant difference (p<0.05) from vehicle group while (**) indicate statistically significant difference from cisplatin-treated group. Statistical analyses among groups were tested using one-way analysis of variance (ANOVA). **(B)** UB/OC-1 cells were treated with vehicle or EGCG (100 µM) for 30 min followed by cisplatin (2.5 µM) for 24 h. and RNA was isolated using TRI reagent. The expression level of the inflammatory genes *CXCR2, CXCR1, CXCL1, TNFα*, and *COX2* were determined by qRT-PCR, *GAPDH* was used as internal control (housekeeping gene) for gene expression normalization. Cisplatin was increased expression of inflammatory genes while EGCG showed significant protection against cisplatin effects (P<0.0007, F(3,16)=11.1 and DF=19). Data were presented fold change mean ± SEM (n≥3). **(C)** UB/OC-1 cells were treated with vehicle or celecoxib (50 µM) for 30 min followed by cisplatin (2.5 µM) for 24 h, RNA was isolated using TRI reagent. The expression level of the inflammatory genes *CXCR2, CXCR1, CXCL1, TNFα* and *COX2* were determined by qRT-PCR, *GAPDH* was used as internal control (housekeeping gene) for gene expression normalization. Cisplatin was increased expression of inflammatory genes while Celecoxib showed significant protection against cisplatin effects (P<0.0001, F(3,16)=80.1 and DF=19). Data were presented fold change mean ± SEM (n≥3). Asterisks, (*) indicates significant difference (p<0.05) from vehicle group, while (**) indicate significant difference (p<0.05) form cisplatin group. Statistical significance of differences among groups were tested using one -way analysis of variance (ANOVA).

In an organ of Corti-derived cell line, UB/OC-1, which has previously been used in auditory research (50), we examined whether cisplatin could also regulate inflammatory genes in these cells and whether SB225002 could directly influence this action. Treatment with cisplatin for 24 h significantly increased the expression of *CXCR2*, *CXCR1*, *CXCL1*, *TNF-α* and *COX2* by 2.9 ± 0.1, 1.9 ± 0.2 and 4.1 ± 0.2-fold, 2.7 ± 0.1 and 1.6 ± 0.1-fold, respectively. The expression of these genes was significantly reduced in cells pretreated with SB225002 (300 nM), suggesting tonic activation of CXCR2 by its endogenous ligands (such as CXCL1 and IL-8) in presence of cisplatin. This concentration of SB225002 did not affect the viability of these cells, measured by the MTS assay (data not shown). The levels of these genes by cisplatin in presence of SB225002 were 1.3 ± 0.2, 1.2 ± 0.1, 2.6 ± 0.1, 1.1 ± 0.1 and 0.5 ± 0.03-fold, respectively, for *CXCL1*, *CXCL2*, *CXCR2*, *TNF-α* and *COX2* (**Figure 11A**). These data indicate that in this cell line, tonic activation of CXCR2 exists, which serves a positive feedback loop for regulation of these chemokines/chemokine receptors and other inflammatory mediators.

Previous studies have demonstrated that noise exposure induces expression of NF-κB in the lateral wall of the cochlea (51). NF-κB and STAT1 up-regulate *CXCL1* gene expression, while knockdown of *STAT1* by siRNA resulted in down-regulation of *CXCL1* and *CXCL2* genes (52). We have previously shown that EGCG down-regulates *STAT1* gene expression in the cochlea and UB/OC-1cells along with pro-inflammatory genes (44). Therefore, we predicted that EGCG could also regulate the expression of these chemokines/chemokine receptors. We show that cisplatin significantly increased the expression of *CXCR2*, *CXCR1*, *CXCL1 TNFα* and *COX2* by 2.8 ± 0.3, 1.8 ± 0.1, 3.5 ± 0.1, 2.7 ± 0.1 and 1.9 ± 0.2-fold, respectively, compared to the vehicle group. In UB/OC-1 cells pretreated with EGCG, the cisplatin-induced expression of these genes was significantly decreased. The fold expression was 0.5 ± 0.2, 0.8 ± 0.1, 1.8 ± 0.1, 0.6 ± 0.1 and 0.9 ± 0.1 respectively (**Figure 11B**). These results indicate a possible role of STAT1 in the induction of these inflammatory genes and suggest that the otoprotective actions of EGCG observed previously derives, at least in part, by suppressing the expression of these immune regulatory genes.

### Role of COX2 in the regulation of *CXCR2* genes

Prostaglandin E2 (PGE2) is produced by cyclooxygenases, COX1 and 2, which represent constitutive and inducible isoforms of this enzyme. PGE2 is released in response to cellular stress (53) and serves as an autocrine/paracrine factor to regulate various cellular functions, such as cell proliferation, immunosuppression and survival (54). We previously showed that cisplatin increased the expression of *COX2* in the cochlea (44, 50). Studies have shown that PGE2 serves as an inhibitory damage-associated molecular patterns (DAMP) to reduce the extent of inflammation induced by trauma and cell apoptosis (53). We examined the possibility that PGE2 serves as a DAMP which is released early in the cochlea in response to cisplatin and which then initiates the expression of *CXCR2*, *CXCR1*, *CXCL1, TNFα* and *COX2* genes. For these studies, we treated UB/OC-1 cells with celecoxib, a COX2 inhibitor, before administering cisplatin. Exposure of UB/OC-1 cells to celecoxib reduced the expression of *CXCR2*, *CXCR1*, *CXCL1, TNFα* and *COX2* to 0.6 ± 0.2, 0.8 ± 0.3, 0.7 ± 0.1, 0.8 ± 0.1 and 1.0 ± 0.1 respectively (**Figure 11C**), compared to the cisplatin-treated group which were 1.8 ± 0.1, 1.6 ± 0.2, 1.5 ± 0.1, 1.9 ± 0.2 and 1.9 ± 0.1, respectively, suggesting that PGE2 (released from cochlear cells as a DAMP) could serve as an early regulator of chemokine-induced inflammation in the cochlea. Interesting, PGE2 has been shown to activate STAT1 serine phosphorylation to enable maturation of dendritic cells (55), suggesting a potential pathway for induction of chemokines/chemokine receptors.

## Discussion

This study provides evidence implicating CXCR2 in cisplatin-induced hearing loss. We show that this chemokine/receptor system is rapidly activated in the cochlea following cisplatin administration orchestrating the events which culminate into cochlear inflammation, pathology and hearing loss. Furthermore, inhibition of CXCR2, or reducing its expression, protected against cisplatin-induced inflammation, pathology and hearing loss. An important finding from this study is that CXCR2-mediated processes are linked to the loss of OHCs and IHC ribbon synapses, as inhibition and/or knockdown of this receptor mitigates synaptopathy and hearing loss. CXCR2 appears to play a permissive role in the cochlear inflammatory process, since inhibition of this receptor attenuated the expression of inflammatory mediators, such as *TNF-α*, *iNOS* and *COX2*, and reduced the levels of CD68 and CD45 positive immune cells detected in the cochlea. Importantly, CXCR2 activation positively regulates the production of CXCL1 in the cochlea. Overall, these studies highlight CXCR2 as novel therapeutic targets for treating cisplatin toxicity.

CXCL1 the major chemoattractant involved in the recruitment of neutrophils to the site of injury (48). This chemokine can bind to the chemokine receptor, CXCR2, present on migrating neutrophils and direct these immune cells to the appropriate target (56). Resident tissue macrophages are the major source of these chemokines, which are synthetized in response to their activation (48). We show that these chemokines are induced early (within 6 h) of cisplatin administration and precede the onset of cochlear pathology and hearing loss (usually evident by 24 h, data not shown). These temporal changes are consistent with the induction of *CXCL1* by resident macrophages which stimulate the subsequent recruitment of neutrophils into the cochlea from the peripheral circulation. A similar temporal profile of induction of *CXCL1* in the hippocampus was observed following administration of LPS (57) and in the spinal cord following paclitaxel treatment in mice (58). CXCL1 immunoreactivity was increased in non-immune cells such as SGs, OHCs and Deiters’ cells which could serve as additional sources of this chemokine for attracting immune cells.

Cisplatin administration induced damage to the cochlea especially in the basal region, which progressed to the apex. We observed reductions in OHC counts in the basal region of cochlea which were attenuated by trans-tympanic administration of agents targeting CXCR2, either the antagonist SB225002 or siRNA against *CXCR2*. The benefits achieved following administration of these agents support the utility of this drug treatment route for delivering therapeutics to the inner ear and suggest that these molecules can enter the cochlea *via* the round window and scala tympani. These benefits are achieved through suppression of the inflammatory component of cisplatin’s cellular actions which involves STAT1-dependent processes (45). While the SV is a site of increased inflammation induced by cisplatin, the present study did not address the possibility that dysfuntion of the stria contributed to hearig loss. We hope to address this issue in a future study. It is not clear what the potential immune cell targets for mediating the anti-inflammatory actions of these agents are. One potential target are infiltrating neutrophils and macrophages whose migration into the cochlea might be reduced by blockade of their homing receptor CXCR2 by the antagonist. This possibility is supported by our data showing that SB225002 blocked the early entry of immune cells (within 24 h) into the cochlea *via* the stria vascularis. Another potential target is resident immune and non-immune cells of the cochlea which express CXCL1 early following cisplatin treatment. Blockade of CXCR2 on immune cells should impede their ability to enter the cochlea. CXCR2 is expressed on resident macrophages which are distributed among the spiral ligament fibrocytes and SG (59). These cells serve as key sentinels of tissue injury and are activated by stimulation of immune receptors such as toll-like receptors, Fc receptors and scavenging receptors present on their cell membranes (60). CXCL1 is expressed by pericytes, perivascular macrophages and perivascular mast cells which could provide the earliest signal for neutrophil migration into the cochlea. As such, inhibition of CXCR2 in the vascular and perivascular space in the strial vasculature is expected to reduce transcytosis of immune cells across the vascular endothelium into the spiral ligament and thereby reduce the migration of immune cells derived from the peripheral circulation into the cochlea. Increases in CXCL1 were observed in these regions within 24 h of cisplatin administration and persisted over the 72h observation period. An interesting finding is that the levels of CXCL1 are also increased in the spiral ganglion neurons and auditory nerve fibers, which persisted over 72 h. This labeling is likely intrinsic to neuronal cells in these regions and could represent a stress marker to stimulate migration of immune cells to the site, as described previously (58).

The trigger for the induction of CXCL1 and immune cell migration could be danger associated molecular patterns (DAMPs), such as high mobility group box 1 protein HMGB1 and S100 proteins, which are rapidly released from stressed or damaged cells (61). HMGB1 can initiate macrophage activation by interacting with cells surface receptors, such as receptor for advanced glycation end products (RAGE), TLR2 and TLR4 (62), and by activating nuclear factor kappa B (NF-κB) and STAT1 transcription factors (63). Another potential DAMP is ATP (64) (65), which is released by stressed or damaged cells in the cochlea. ATP could act on cell surface receptors (such as P2X7) on resident cells to stimulate release of inflammatory cytokines and chemokines in order to initiate and maintain the inflammatory response (66). We speculate that the release of CXCL1 is one of these early responses produced by engagement of ATP with cell surface purinergic receptors, such as P2X7 receptors. P2 receptors are on resident immune cells and on sensory and non-sensory epithelia of the OC, SG and SV where they could protect the cochlea from overstimulation but could also initiate apoptotic and repair processes (67). ATP is rapidly metabolized to adenosine through the action of cell surface ectonucleotidases (such as CD39 and CD73). Adenosine could protect the cochlea by suppressing the activation of resident macrophages (68) in addition to protecting the cochlea through its antioxidant properties (50).

An interesting finding derived from this study is that CXCR2 is tonically active, as knockdown or inhibition of this receptor significantly reduced the expression of some cytokines and inflammatory mediators. This activation could reflect activation of the receptor by CXCL1 present in the extracellular environment under normal conditions, in addition to the contribution of IL-8, another activator of CXCR2. Tonic activation of the receptor could also lead to tonic suppression of *STAT3* expression, which was significantly increased by inhibition or knockdown of CXCR2.

Our data show that cisplatin stimulates the expression of prostaglandin E2 (PGE2), a factor which also serves as an inhibitory DAMP released from damaged of dying cells (53). We showed that cisplatin can increase *PGE2* expression *in vitro via* a CXCL1/CXCR2 pathway, which in turn, can positively regulate the expression of *CXCL1*, *CXCR1* and *CXCR2*. Interestingly, while celecoxib inhibits COX2 and decreases PGE2 production it also decreased *PGE2* expression and the expression of *CXCL1*, *CXCR1* and *CXCR2*, indicating that these factors are positively regulated by PGE2 in the cochlea. However, PGE2 can also serve additional (beneficial) roles in the immune system, as it has been shown to promote a regulatory type (M2) phenotype in macrophage by stimulating protein kinase A-salt-inducible kinase (SIK)-CREB-regulated transcription coactivator (CRTC)3 (69).

CXCR2 ligation appears essential for mediating cisplatin-induced decrease of IHC ribbon synapses, leading to reduction in wave I amplitude and latency prolongation. This likely involves increased localized inflammatory response in the cochlea. We have previously shown that EGCG, which possesses both antioxidant and anti-inflammatory properties, can protect against cisplatin induced-hearing loss and IHCs synaptopathy (44). Similarly, activation of cannabinoid 2 (CB2) receptor, linked to antioxidant and anti-inflammatory actions in the cochlea, protects against cisplatin-induced cochlear synaptopathy (70), linking these properties in the regulation of ribbon synapses. It is possible that “protective” treatments could regulate the function of classical immune cells such as astrocytes or microglia which target synapse loss through the process of synaptic pruning. In the central nervous system, this process could require the additional involvement of components of the complement system. For example, the elimination of retinal ganglion cells inputs into the brain by microglia is dependent on neural activity and complement receptor 3 and its ligand C3 (71). Synapse elimination is also dependent on transforming growth factor (TGF-β) and the expression of phagocytic receptors, such as mer tyrosine kinase (MERTK) and multiple EGF like domains 10 (MEGF10) (72). In addition, synapses with reduced activity such as those possessing silent α-amino-3-hydroxy-5-methyl-4-isoxazolepropionic acid (AMPA) receptors are also tagged for elimination (73). The availability of trophic support could also contribute to synaptic integrity. Acoustic trauma induced by noise or aminoglycoside produces a deficit in neurotrophin-3 (NT-3) and brain-derived neurotrophic factor (BDNF) which are important for maintaining synaptic integrity. Administration of NT-3 stimulates synapse regeneration *in vivo* (74, 75) and *in vitro* (76). We speculate that, like aminoglycoside, cisplatin could reduce NT-3 and BDNF levels and thereby promote loss of ribbon synapses. In this regard, NT-3 has been shown to reduce cisplatin-induced damage to SG neurons (77). The finding that blockade of CXCR2 protected against synaptic loss suggests that inflammation might reduce the levels of the trophic factors at these synapses (78, 79). Inflammation-induced synaptic loss has been previously described (80).

**Figure 12:**
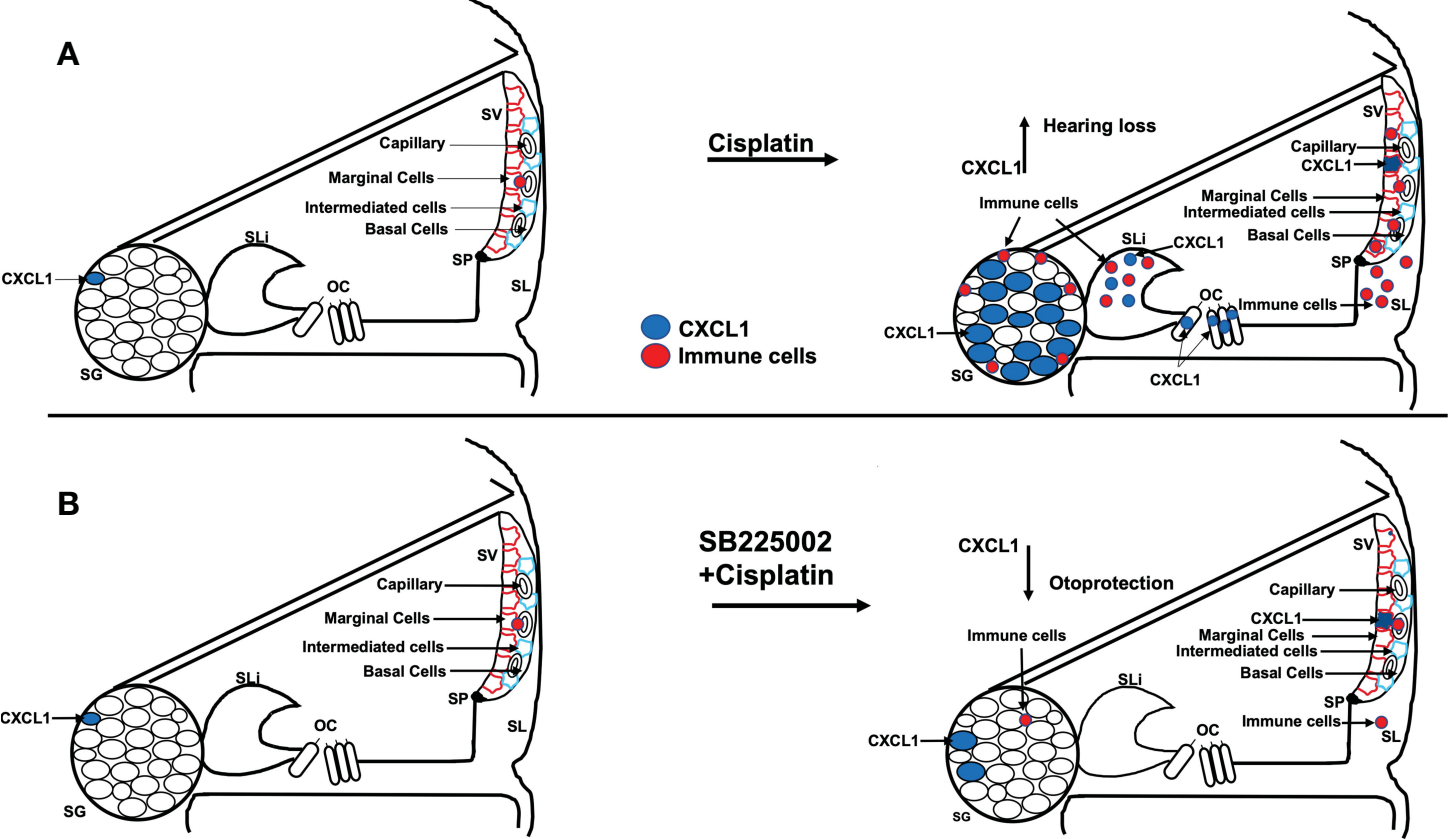
Scheme show the hypothesis of cisplatin effect on cochlea. **(A)** Cisplatin administration up regulates CXCL1 levels, especially in SG, and recruit immune cells migration to cochlea through spiral ligament and stria vascularis. Pretreatment with trans-tympanic SB225002 **(B)** followed by cisplatin administration down regulates CXCL1 expression in SG neurons, SV and SL and inhibit immune cell migration to cochlea.

In summary, we show that the CXCL1 is important for the early activation of pro-inflammatory signaling leading to cisplatin-induced hearing loss. Cisplatin administration increases in CXCL1 mainly in the organ of Corti, spiral limbus and spiral ganglion neurons. Inhibition of this pathway reduced immune cell migration through the stria vascularis and spiral ligament and reduced cisplatin-induced synaptopathy and hearing loss (see **Figure 12**). Thus, inhibiting CXCR2 could serve as a novel method for treating cisplatin-induced hearing loss.

## Data availability statement

The original contributions presented in the study are included in the article/**Supplementary Material**. Further inquiries can be directed to the corresponding author.

## Ethics statement

The animal study was reviewed and approved by Division of Laboratory Animal Medicine (DLAM) facility of SIU School of Medicine.

## Author contributions

RA and VR conceived the idea for the research presented in this article. RA, EA and IA planned and performed the experiments and assisted with data analysis. RA and VR wrote the main manuscript text and edited all figures. DA, SS, SP, AO and LR critiqued and revised the manuscript. All authors reviewed and approved the manuscript. All authors contributed to the article and approved the submitted version.

## Supplementary material

Supplementary Figure 1 Quantification of image intensities for . Immunolabeling for CXCL1 was analyzed by Image J and presented as the normalized intensities versus vehicle-treated cochlea (p < 0.05, N=4) using one-way ANOVA.

Supplementary Figure 2 Quantification of image intensities for . **(A)** CD68 labeling was enhanced by cisplatin treatment for 24 h and was progressively increased over a 72 h period. **(B)** CD45 labeling increases starting at 24 h and becoming more intense by 72 h. Protein expression quantity for CD68 and CD45 were analyzed by Image J and presented as the normalized intensities versus vehicle-treated cochlea. (p < 0.05, N=4) using one-way ANOVA. Asterisk (*) indicate statistically significant from vehicle (p < 0.05, N=4).

Supplementary Figure 3 Quantification of image intensities for . **(A, B)** CXCL1 and CD68 protein expression levels were increased by cisplatin treatment, whereas SB225006 abolished the effect of cisplatin. The levels of CXCL1 and CD68 were analyzed by Image J and presented as the normalized intensities versus vehicle-treated cochlea. **(C, D)**, CD45 and IBA1 immunolabeling were increased in SG and SVA following cisplatin treatment. The levels of these marker were decreased in the animals pretreated with SB225002, followed by cisplatin. Asterisk (*) indicate statistically significant from vehicle (p < 0.05, N=4) while (**) indicate statistically significant difference from cisplatin-treated group (p < 0.05, N=4) using one-way ANOVA.

Supplementary Figure 4 Trans-tympanic delivery of SB225002 attenuated cisplatin ototoxicity. Wistar rats were administration trans-tympanically with vehicle or SB225002 (1.4 nmoles/ear) and cisplatin (11mg/kg) for 24 hr. **(A)** Basal, middle was stained with Myosin VIIa (magenta) to visualize OHCs and IHCs. Representative whole-mount images showed no damage of OHCs or IHC by cisplatin. Scale bar represent 20 µm. **(B)** Cochlear basal turn sections were stained with myosin VIIa (blue), CtBP2 pre-synaptic marker (red) and GluR2 post-synaptic marker (green). Representative whole-mount images from turns showed that cisplatin reduced the number of ribbon synapse per IHC, whereas these effects was abolished by SB225002. Orphan synapses were depicted as staining with either GluR2 or CtBP2 alone, but not both (indicated by white arrows). Scale bar represent 10 µm. **(C)** Graph shows the number of synaptic ribbons per IHC which were substantially preserved by SB225002. **(D)** Cisplatin significantly induced orphan synapses per IHC which were reduced by SB225002. Data are presented as the mean ± SEM of four animals. Asterisks, (*) indicates significant difference (p<0.05) from vehicle group, while (**) indicate significant difference (p<0.05) form cisplatin group.

## Glossary

**Table:**

ABR	Auditory brainstem response
AMPA	α-Amino-3-hydroxy-5-methyl-4-isoxazolepropionic acid
ANOVA	Analysis of variance
ATP	Adenosine Tri phosphate
BDNF	brain-derived neurotrophic factor
CB2	Cannabinoid 2
CCL2	Chemokine (C-C motif) ligand 2
CCL4	Chemokine (C-C motif) ligands 4
CD39	Ectonucleoside triphosphate diphosphohydrolase-1
CD45	Cluster of differentiation 45 (lymphocyte common antigen)
CD68	Cluster of differentiation 68
CD73	Ecto-5′-nucleotidase
COX-2	Cyclooxygenase-2
CtBP2	C-terminal binding protein 2
CXCL1	Chemokine (C-X-C motif) ligand 1
CXCL2	Chemokine (C-X-C motif) ligand 2
CXCL12	Chemokine (C-X-C motif) ligand 12
CXCR1	Chemokine (C-X-C motif) receptor 1
CXCR2	Chemokine (C-X-C motif) receptor 2
CX3CR1	Motif chemokine receptor 1
DAB	3,3’-diaminobenzidine
DAMP	Damage-associated molecular patterns
dB	Decibel
EGCG	Epigallocatechin-3-gallate
GAPDH	Glyceraldehyde-3 phosphate dehydrogenase
GluR2	Glutamate receptor 2
GRO	human growth-regulated oncogene
HMGB1	High mobility group box 1 protein
IBA1	ionized calcium-binding adapter molecule 1
IL6	Interleukin-6
IL10	Interleukin-10
iNOS	Inducible nitric oxide synthetase
IHC	Inner hair cells
KC	Keratinocyte chemoattractant
LDL	Oxidized low-density lipoprotein
MERTK	Mer Tyrosine Kinase
MEGF10	Multiple EGF Like Domains 10
MIP-2	Macrophage inflammatory peptide 2
MTS	(3-(4,5-dimethylthiazol-2-yl)-5-(3-carboxymethoxyphenyl)-2-(4-sulfophenyl);2H-tetrazolium)
Myo VIIa	Myosin VIIa
NT-3	Neurotrophin-3
NOX-3	NADPH oxidase 3
NF-κB	Nuclear factor kappa B
OC	Organ of Corti
OHC	Outer hair cells
P2X7	P2X purinoceptor 7
PGE2	Prostaglandin E2
PBS –	Phosphate Buffer Saline
RAGE	Receptor for Advanced Glycation Endproducts
ROS	Reactive oxygen species
sICAM-1	Soluble intercellular adhesion molecule-1
SLi	Spiral limbus
SEM	Standard error of mean
SG	Spiral ganglion neuron
SL	Spiral ligament
SPL	Sound pressure level
STAT1	Signal transducer and activator of transcription 1
STAT3	Signal transducer and activator of transcription 3
TGF-β	Transforming growth factor
SVA	Stria vascularis
TNF-α	Tumor necrosis factor alpha
TLRs	Toll-like receptors
TLR2	Toll-like receptor 2
TLR4	Toll-like receptor 4
UB/OC-1	University Bristol/Organ of Corti-1
VEGF	Vascular endothelial growth factor
