# Supplementary figures and images for: Targeting CXCL1 chemokine signaling for treating cisplatin ototoxicity
Source: Front Immunol. 2023 Mar 31;14:1125948. doi: 10.3389/fimmu.2023.1125948 (PMC10102581; doi:10.3389/fimmu.2023.1125948)

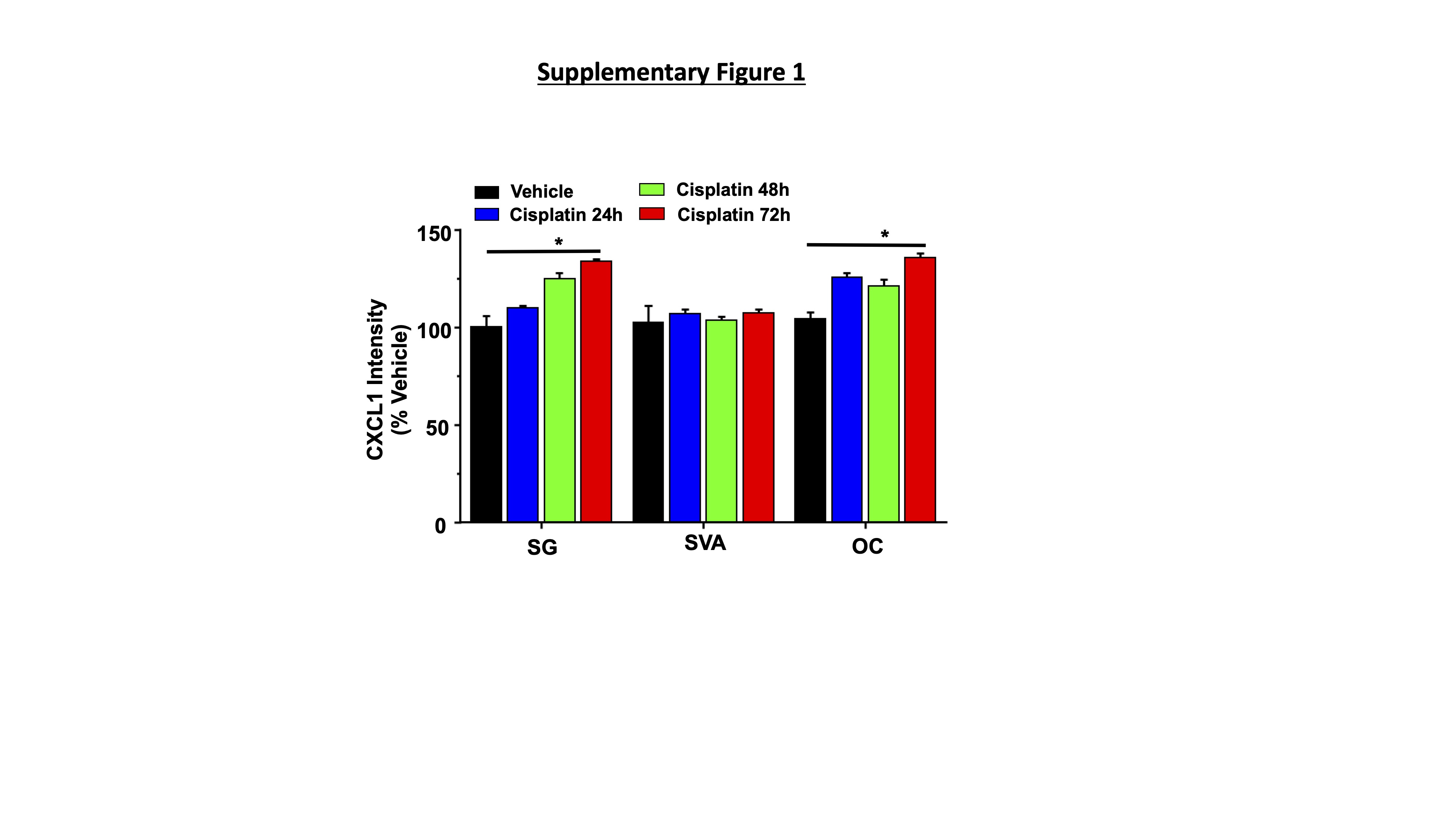

Supplement: Supplementary Figure 1 — Quantification of image intensities for . Immunolabeling for CXCL1 was analyzed by Image J and presented as the normalized intensities versus vehicle-treated cochlea (p < 0.05, N=4) using one-way ANOVA. [file Image_1.jpg]

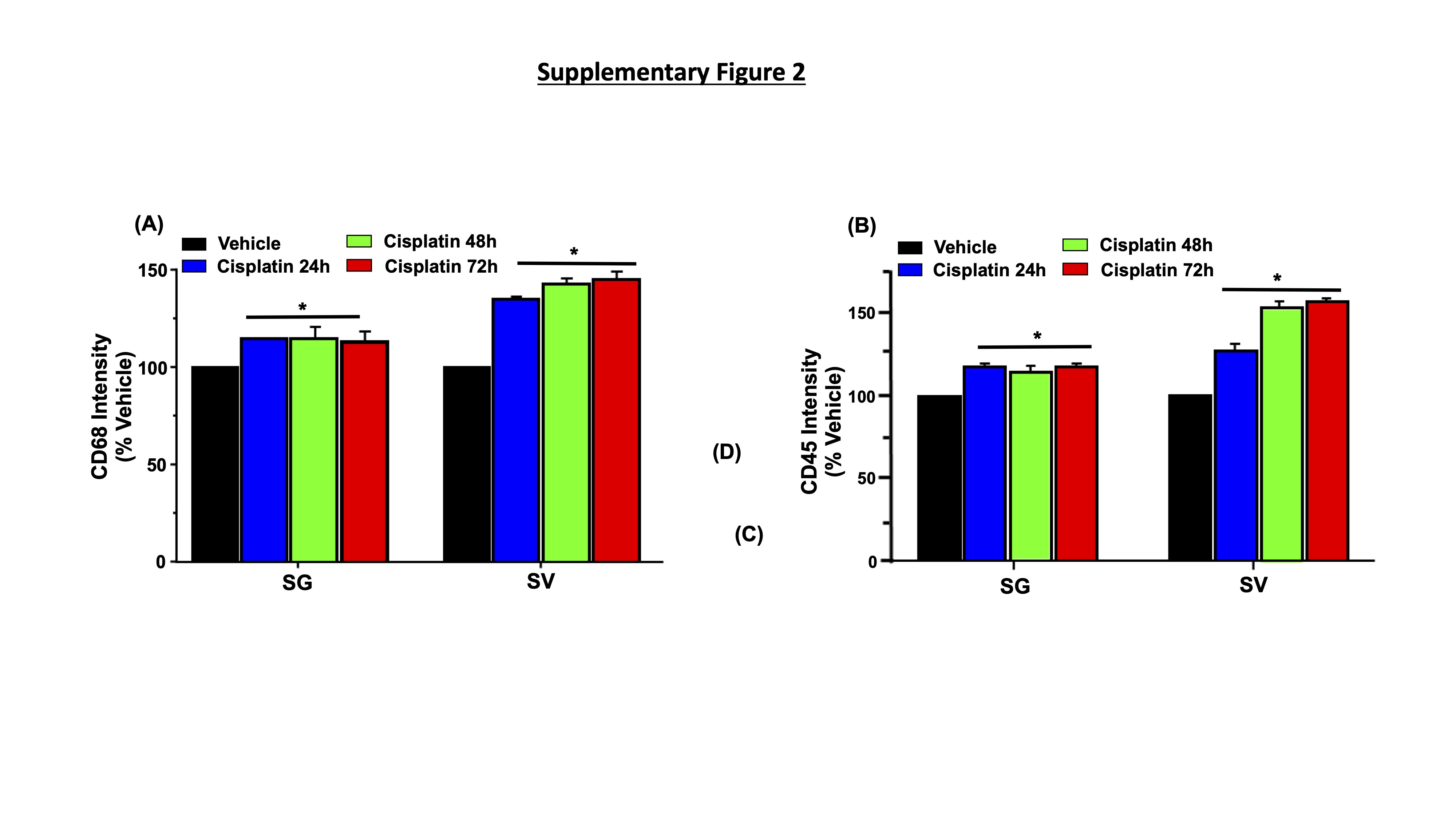

Supplement: Supplementary Figure 2 — Quantification of image intensities for . (A) CD68 labeling was enhanced by cisplatin treatment for 24 h and was progressively increased over a 72 h period. (B) CD45 labeling increases starting at 24 h and becoming more intense by 72 h. Protein expression quantity for CD68 and CD45 were analyzed by Image J and presented as the normalized intensities versus vehicle-treated cochlea. (p < 0.05, N=4) using one-way ANOVA. Asterisk (*) indicate statistically significant from vehicle (p < 0.05, N=4). [file Image_2.jpg]

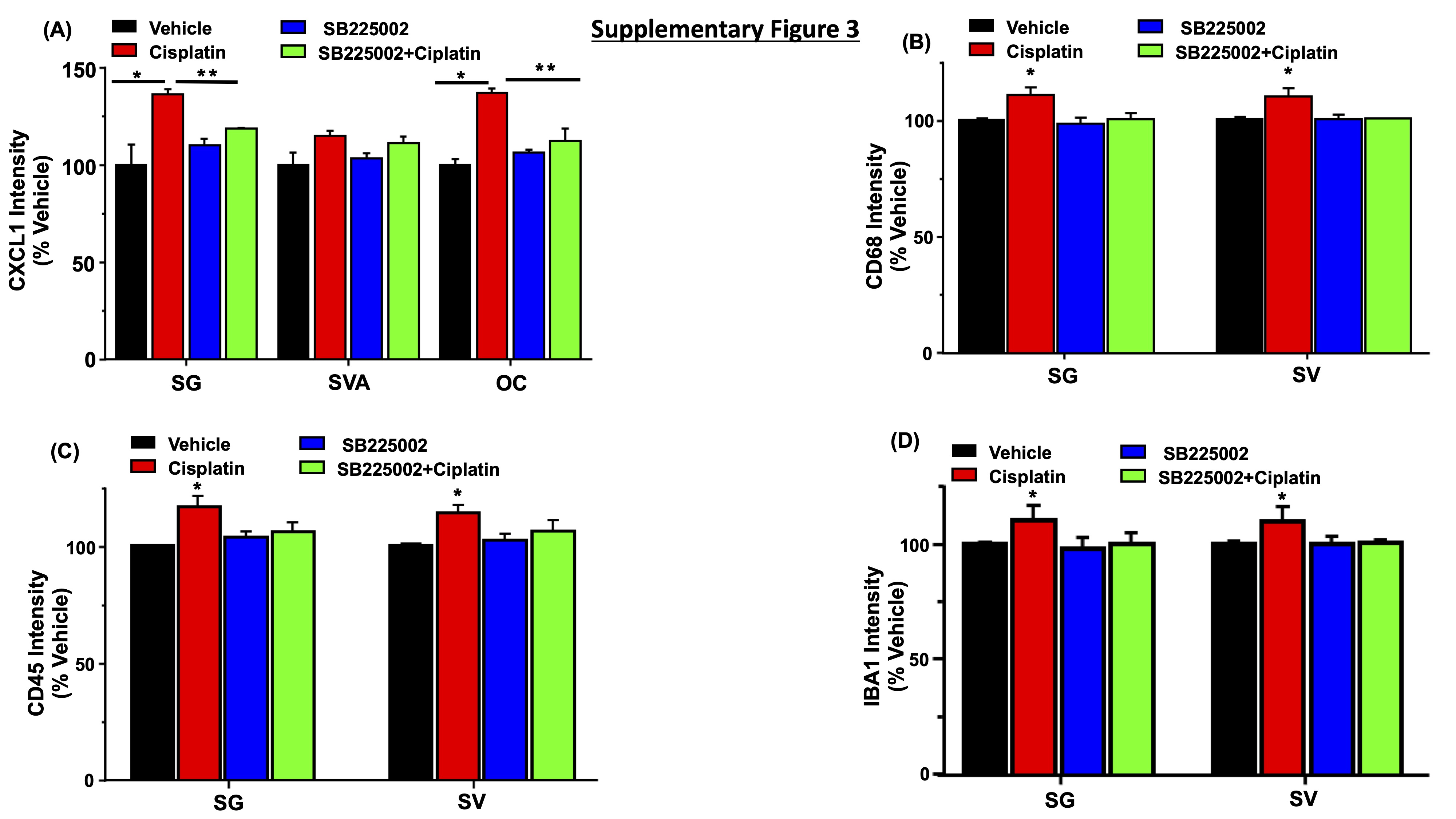

Supplement: Supplementary Figure 3 — Quantification of image intensities for . (A, B) CXCL1 and CD68 protein expression levels were increased by cisplatin treatment, whereas SB225006 abolished the effect of cisplatin. The levels of CXCL1 and CD68 were analyzed by Image J and presented as the normalized intensities versus vehicle-treated cochlea. (C, D), CD45 and IBA1 immunolabeling were increased in SG and SVA following cisplatin treatment. The levels of these marker were decreased in the animals pretreated with SB225002, followed by cisplatin. Asterisk (*) indicate statistically significant from vehicle (p < 0.05, N=4) while (**) indicate statistically significant difference from cisplatin-treated group (p < 0.05, N=4) using one-way ANOVA. [file Image_3.jpg]

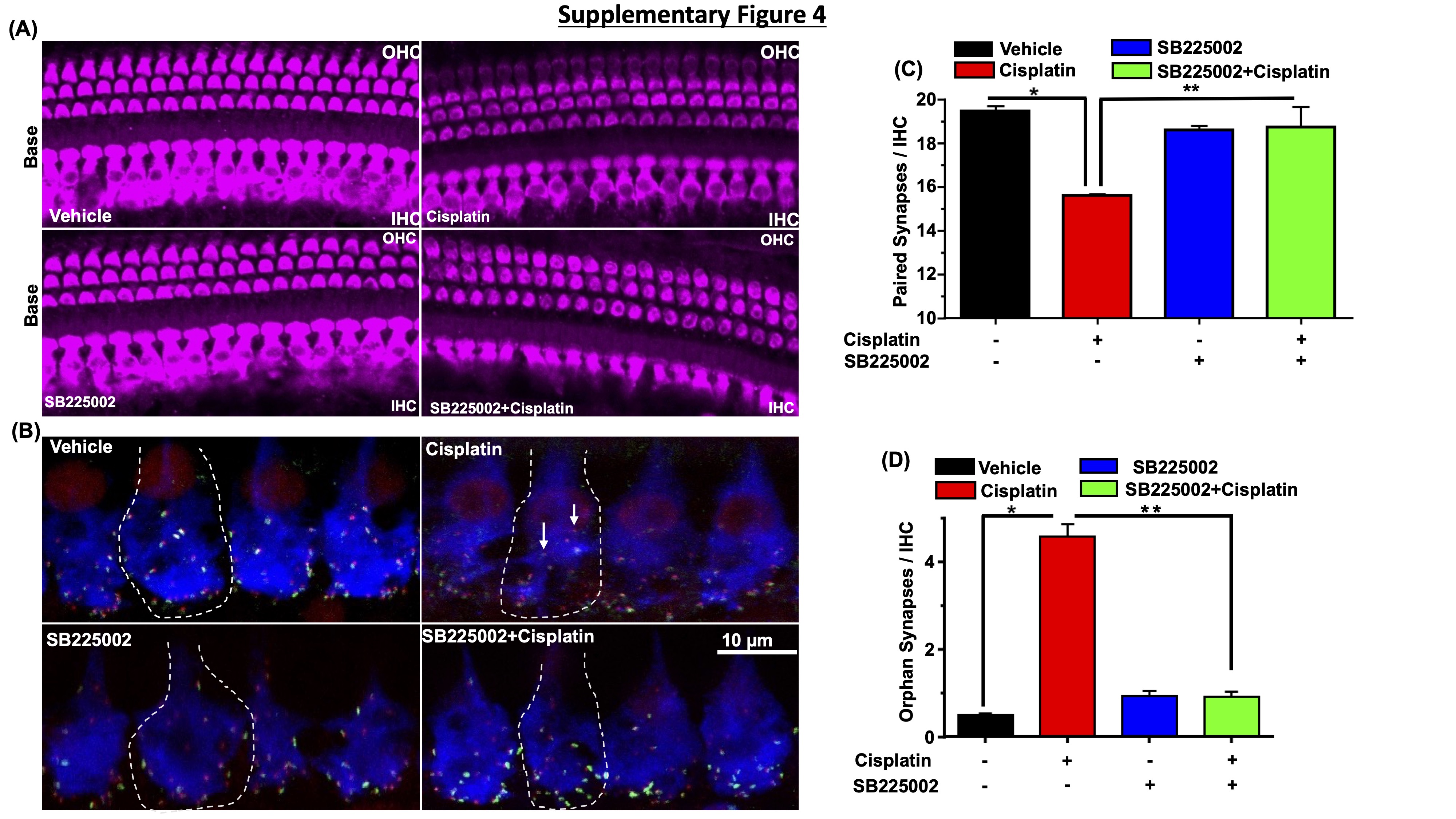

Supplement: Supplementary Figure 4 — Trans-tympanic delivery of SB225002 attenuated cisplatin ototoxicity. Wistar rats were administration trans-tympanically with vehicle or SB225002 (1.4 nmoles/ear) and cisplatin (11mg/kg) for 24 hr. (A) Basal, middle was stained with Myosin VIIa (magenta) to visualize OHCs and IHCs. Representative whole-mount images showed no damage of OHCs or IHC by cisplatin. Scale bar represent 20 µm. (B) Cochlear basal turn sections were stained with myosin VIIa (blue), CtBP2 pre-synaptic marker (red) and GluR2 post-synaptic marker (green). Representative whole-mount images from turns showed that cisplatin reduced the number of ribbon synapse per IHC, whereas these effects was abolished by SB225002. Orphan synapses were depicted as staining with either GluR2 or CtBP2 alone, but not both (indicated by white arrows). Scale bar represent 10 µm. (C) Graph shows the number of synaptic ribbons per IHC which were substantially preserved by SB225002. (D) Cisplatin significantly induced orphan synapses per IHC which were reduced by SB225002. Data are presented as the mean ± SEM of four animals. Asterisks, (*) indicates significant difference (p<0.05) from vehicle group, while (**) indicate significant difference (p<0.05) form cisplatin group. [file Image_4.jpg]
